# A refined MASH–HCC model identifies macrophage Gadd45b as a key orchestrator of inflammation-driven neoplastic progression

**DOI:** 10.1038/s12276-026-01838-5

**Published:** 2026-09-16

**Authors:** Hyunmi Kim, Gyeonghun Kim, Hyojin Yeon, Da-Yeon Yang, Sang Gyu Lee, Tae Hyeon An, Seung Yeon Oh, Seona Yoon, Jiwoo Kim, Juyong Choi, Hyun-Ju Park, Eun-Woo Lee, Baek-Soo Han, Chul-Ho Lee, Il Yong Kim, Won Kon Kim, Kwang-Hee Bae, Jun Won Park, Seung Hyun Oh, Young Nyun Park, Murim Choi, Je Kyung Seong, Kyoung-Jin Oh

**Affiliations:** 1 Metabolic Regulation Research Center, Korea Research Institute of Bioscience and Biotechnology, Daejeon, Republic of Korea; 2 Laboratory of Developmental Biology and Genomics, College of Veterinary Medicine, Seoul National University, Seoul, Republic of Korea; 3 Korea Model animal Priority Center, Seoul National University, Seoul, Republic of Korea; 4 Department of Biomedical Sciences, Seoul National University College of Medicine, Seoul, Republic of Korea; 5 Department of Functional Genomics, KRIBB School of Bioscience, Korea University of Science and Technology, Daejeon, Republic of Korea; 6 Interdisciplinary Program in Bioinformatics, Seoul National University, Seoul, Republic of Korea; 7 Biodefense Research Center, Korea Research Institute of Bioscience and Biotechnology, Daejeon, Republic of Korea; 8 Laboratory Animal Resource Center, Korea Research Institute of Bioscience and Biotechnology, Daejeon, Republic of Korea; 9 Laboratory Animal Medicine, College of Veterinary Medicine, Seoul National University, Seoul, Republic of Korea; 10 Laboratory of Veterinary Histology, College of Veterinary Medicine, Seoul National University, Seoul, Republic of Korea; 11 Department of Pathology, Severance Hospital, Yonsei University College of Medicine, Seoul, Republic of Korea; 12 Interdisciplinary Program of Cancer Biology, Seoul National University Cancer Research Institute, Seoul, Republic of Korea; 13 BIO MAX/N-Bio Institute, Seoul National University, Seoul, Republic of Korea

**Keywords:** Obesity, Mechanisms of disease, Metabolic disorders

## Abstract

Metabolic dysfunction-associated steatohepatitis (MASH) is emerging as a leading driver of hepatocellular carcinoma (HCC), yet the molecular mechanisms linking metabolic stress, chronic inflammation and tumorigenesis remain poorly understood. Here we established a metabolically relevant, time-efficient MASH-to-HCC model in C57BL/6N mice by combining a MASH diet with controlled CCl_4_ administration, enabling stepwise recapitulation of MASH-associated neoplastic progression. Using this model, we identified growth arrest and DNA damage 45b (Gadd45b) as a novel MASH-derived protumorigenic regulator selectively activated under metabolic stress. Integrated analyses of human bulk and single-cell transcriptomic datasets and mouse transcriptomic deconvolution revealed concordant macrophage remodeling and *GADD45B/Gadd45b* expression dynamics during MASH-to-HCC progression. Mechanistically, fatty acids and TNFα preferentially induced Gadd45b in macrophages, where it amplified TNFα–NF-κB signaling. Macrophage-derived inflammatory signals subsequently induced Gadd45b and NF-κB activation in hepatocytes, establishing a feed-forward inflammatory loop that promoted fibrogenic and partial EMT-like programs and tumor spheroid formation. Importantly, temporal profiling during spheroid formation and progression revealed transient induction of Gadd45b during early spheroid establishment, but not during later progression, indicating that Gadd45b-mediated inflammatory signaling primarily promotes tumor initiation rather than subsequent growth. Consistent with human data, *Gadd45b* expression increased with disease severity and positively correlated with inflammatory factors in the MASH–HCC model, whereas pharmacological inhibition attenuated the Gadd45b–inflammation signaling axis. Collectively, our findings establish macrophage Gadd45b as a key orchestrator linking metabolic stress, chronic inflammation, and neoplastic transformation during MASH-to-HCC progression. Our refined MASH–HCC model provides a robust platform for mechanistic studies and preclinical evaluation of inflammation-targeted therapies.

## Macrophage Gadd45b links metabolic stress to liver cancer

Hepatocellular carcinoma (HCC) is increasingly linked to metabolic dysfunction-associated steatohepatitis (MASH), a severe form of fatty liver disease. This study addresses the unclear mechanisms driving MASH to HCC transition. Researchers developed a refined MASH-HCC mouse model using C57BL/6N mice fed a MASH-provoking diet combined with carbon tetrachloride. This approach enables relatively rapid HCC development under moderate fibrotic stress, limiting excessive CCl_4_-driven fibrosis while more physiologically recapitulating diet-driven MASH–HCC progression. The model enables the identification of Gadd45b as a key protumorigenic regulator, linking chronic inflammation to liver cancer development. Gadd45b amplifies TNFα–NF-kB signaling, promoting fibrosis and tumor formation. This model and findings provide a platform for studying inflammation-driven liver cancer and evaluating anti-inflammatory therapies. Future research could explore Gadd45b-targeted interventions to prevent MASH-associated HCC progression. This summary was initially drafted using artificial intelligence, then revised and fact-checked by the author.

## Introduction

Hepatocellular carcinoma (HCC) is one of the most lethal and fastest-growing cancers worldwide, traditionally linked to chronic viral hepatitis^1^. However, with the global surge in obesity and metabolic syndrome, the epidemiology of HCC is shifting toward nonviral etiologies—particularly metabolic dysfunction-associated steatohepatitis (MASH), now emerging as a leading cause of liver cancer^2–5^. Despite this growing clinical burden^6,7^, the mechanisms that drive the transition from MASH to HCC remain incompletely understood.

As a progressive form of metabolic dysfunction-associated fatty liver disease (MAFLD), MASH is characterized by hepatic steatosis, lobular inflammation and varying degrees of fibrosis, which together create a proinflammatory microenvironment conducive to neoplastic transformation^8^. While various diet-, toxin-, genetic- or combination-based models have been developed to study MASH-derived HCC, each exhibits distinct limitations, including extended timeframes, inconsistent tumor incidence or nonphysiological, toxin-driven responses rather than metabolically induced disease phenotypes^9–15^. Among these models, short-term models combining a Western diet and carbon tetrachloride (CCl_4_) have been widely used to induce MASH–HCC in mice owing to their time efficiency^16–18^; however, they often result in excessive fibrosis and nonphysiological liver injury, which limits their ability to capture the gradual progression of MASH to HCC.

Recognizing that both mouse strain and dietary composition are critical for balancing metabolic injury and toxin-driven fibrosis, we refined the short-term model by establishing a MASH-provoking diet as an essential driver of MASH-to-HCC progression in C57BL/6N mice, in combination with CCl_4_. Compared with the commonly used C57BL/6J strain^16,17,19–21^, C57BL/6N mice exhibit more efficient hepatic lipid deposition^22,23^ and reduced susceptibility to excessive CCl_4_-induced liver fibrosis, thereby enabling a more gradual and metabolically coordinated progression of liver pathology^18,22,23^. The MASH diet rapidly induces key MASH phenotypes within 16 weeks^24–26^. Compared with previously reported Western diet-based C57BL/6N models requiring up to 48 weeks^18^, we demonstrate that our MASH diet-based C57BL/6N model induced MASH-to-HCC progression within 24 weeks. This substantial reduction in experimental duration enhances the practicality of a mouse model and establishes a rapid, metabolically relevant, inflammation-driven platform for in-depth mechanistic investigation of the MASH-to-HCC transition.

Chronic inflammation is a hallmark of both MASH and HCC^27–30^. In our refined model, transcriptomic analysis revealed TNFα–NF-kB signaling as the most prominently upregulated pathway during MASH-to-HCC progression. While TNFα–NF-kB activation is a common feature of inflammatory diseases, our data identified growth arrest and DNA damage 45b (Gadd45b)^31^ as an effector selectively engaged by metabolic stress in MASH progression. Consistently, analysis of public human bulk RNA sequencing (RNA-seq) datasets revealed a progressive increase in *GADD45B* expression together with inflammatory markers during MASH-to-HCC progression. Single-cell transcriptomic analysis further demonstrated that a disease stage-dependent remodeling of macrophage populations during MASH progression, accompanied by a corresponding shift in *GADD45B* expression across macrophage subsets. Specifically, *GADD45B* expression was elevated in infiltrating macrophages at early stages of MASH and further increased in liver-resident Kupffer cells (KCs) at advanced stages of MASH. These findings build on previous reports that Gadd45b is associated with inflammatory activation in macrophages, while its functional role in macrophages under the metabolically stressed and tumorigenic conditions in MASH remains unaddressed.

Here, we identify Gadd45b as a previously unrecognized protumorigenic regulator linking chronic inflammation to hepatocarcinogenesis in MASH. We found that Gadd45b is predominantly expressed in liver macrophages under MASH conditions and functions as a critical amplifier of TNFα–NF-kB signaling in the liver. Mechanistically, Gadd45b drives a feed-forward proinflammatory circuit through macrophage–hepatocyte crosstalk, thereby promoting fibrosis, epithelial–mesenchymal transition, and tumor spheroid formation—key steps in neoplastic transformation. Collectively, our findings establish Gadd45b as a molecular driver of MASH-to-HCC progression and provide a refined model for dissecting inflammation-driven hepatocarcinogenesis and evaluating targeted anti-inflammatory therapies.

## Materials and methods

### Animal studies

All the 8–9-week-old C57BL/6N mice were purchased from ORIENT BIO and housed and maintained in a 12-h light/12-h dark cycle under temperature- and humidity-controlled conditions with free access to food and water. All animal procedures were approved by the Institutional Animal Care and Use Committee of the Korea Research Institute of Bioscience and Biotechnology (KRIBB-AEC-24298) and were performed in accordance with the Guidelines for the Care and Use of Laboratory Animals published by the US National Institutes of Health.

### Animal study design 1: dietary and CCl_4_ treatment

Nine-week-old male mice were fed either a normal chow diet (NCD) with tap water or a MASH-provoking diet (29% fat, 34% sucrose, 1.25% cholesterol; TD.190142, Envigo) with high-sugar water containing 23.1 g/l d-fructose (F0127, Sigma-Aldrich) and 18.9 g/l d-glucose (G8270, Sigma-Aldrich). The CCl_4_ (289116, Sigma-Aldrich)-treated mice received intraperitoneal injections of either low (0.3 ml/kg) or high doses (0.9 ml/kg) of CCl_4_ in corn oil, or just corn oil as a control. These treatments were administered twice weekly for 24 weeks, starting concurrently with the diet administration. At the end of 24 weeks, the mice were euthanized, and liver and serum samples were collected for histological, serological and gene expression analyses.

### Animal study design 2: *Gadd45b* knockout and knockdown mice

Nine-week-old male *Gadd45b*^−/−^ mice obtained from Dr. Chul-Ho Lee’s Lab at the Korea Research Institute of Bioscience and Biotechnology (KRIBB) were fed a high-fat diet (HFD; 60 kcal% fat diet, D12492, Research Diets) for 10 weeks. For *Gadd45b* knockdown studies, 9-week-old male C57BL/6N mice were fed an HFD for 22 weeks and subsequently administered Ad-sh*Gadd45b* or Ad-US control (0.5 × 10^9^ plaque-forming units per mouse) via tail-vein injection. Mice were euthanized 6 days post-injection to allow sufficient *Gadd45b* knockdown.

### Animal study design 3: HCC development in MASH–HCC male/female mice

Nine-week-old male and female mice were fed a MASH diet with high-sugar water and administered high doses of CCl_4_ for 8, 16, 20 and 24 weeks. At each time point, the mice were euthanized, and serum samples were collected. The livers were examined macroscopically for tumor growth, and the number of tumor nodules was counted and recorded.

### Animal study design 4: drug administration

Mice were fed a MASH diet with high-sugar water and administered high doses of CCl_4_ for 16 or 24 weeks. For the final 8 weeks, they received daily oral administration of vehicle or obeticholic acid (OCA; 30 mg/kg, HY-12222, MCE) dissolved in 0.5% carboxymethyl cellulose.

### qPCR analysis

Total RNA was extracted from mouse tissues and cells using the TRIzol reagent (15596018, Invitrogen), and cDNA was synthesized using M-MLV Reverse Transcriptase (M1705, Promega). Quantitative PCR (qPCR) was performed using the SYBR green PCR kit in a C1000 Touch Thermal Cycler (Bio-Rad Laboratories). All data were normalized to ribosomal L32 in the corresponding sample. The primer sequences are presented in Supplementary Table 1.

### Blood analysis and ELISA

Plasma levels of alanine aminotransferase (ALT), aspartate aminotransferase (AST), total cholesterol and triglyceride were measured using an automated blood chemistry analyzer (AU480, Beckman Coulter). Plasma alpha-fetoprotein (AFP) levels were determined via Mouse AFP ELISA kit (MAFP00, R&D Systems), according to the manufacturer’s protocol.

### Tissue processing and histology analysis

Liver tissues were fixed in 10% neutral-buffered formalin solution (FR2013-000-00, Biosesang), paraffin-embedded and sectioned (~3–5 μm)^32^. For histological analysis, the tissue slides were stained with hematoxylin and eosin (H&E) and scored for steatosis, lobular inflammation and hepatocyte ballooning based on the Nonalcoholic Steatohepatitis Clinical Research Network (NASH CRN) scoring system to evaluate the NAFLD activity score (NAS)^33,34^. To assess hepatic fibrosis, liver sections were stained with picrosirius red (PSR; Ab248632, Abcam) and graded using the Ishak scoring system^33,35,36^. The histological scoring results were reviewed by a pathologist. Reticulin staining was performed using the Reticulum Stain Kit (SSK5037-250, BBC Biochemical) according to the manufacturer’s instructions.

### Immunohistochemistry and immunofluorescence staining

Paraffin sections were deparaffinized, rehydrated and antigen retrieval was performed using antibody-specific solutions. Endogenous peroxidase activity was then quenched with hydrogen peroxide, and blocking was conducted according to each antibody’s protocol. The sections were incubated with primary antibodies overnight at 4 °C, followed by secondary antibodies and 3,3′-diaminobenzidine detection. For immunofluorescence staining, GADD45β (sc-8776, Santa Cruz Biotechnology) was used as the primary target protein in all conditions. To identify specific cell types, costaining with cell-type markers was performed. Slides were incubated with primary antibodies overnight at 4 °C, followed by fluorophore-conjugated secondary antibodies; donkey anti-goat IgG Alexa Fluor 568 (A11057, Thermo Fisher Scientific) and donkey anti-rabbit IgG Alexa Fluor 647 (A31573, Thermo Fisher Scientific). Nuclei were counterstained using an antifade mounting medium containing 4′,6-diamidino-2-phenylindole (DAPI). Fluorescent signals were visualized using confocal microscopy, and representative images were acquired for each staining combination. Additional information on the antibodies used in this study is listed in Supplementary Table 2.

### mRNA sequencing

RNA libraries were prepared with 0.5 μg of total RNA using the Illumina TruSeq Stranded Total RNA Library Prep Gold Kit (#20020599, Illumina) and sequenced (2×100 bp, paired-end) on an Illumina NovaSeq platform (Macrogen). Sequencing data were processed using SAMtools v 1.9 for sorting and indexing, and transcripts were assembled and quantified with StringTie v2.1.3b (Pertea)^37,38^. Gene- and transcript-level quantification was calculated as raw read count, fragments per kilobase of transcript per million mapped reads (FPKM) and transcripts per million (TPM). Differential gene expression analysis was performed by DESeq2 v 1.38.3^39^, with the significant genes were filtered using the criteria |fold change| ≥2 and raw *P* value <0.05. Hierarchical clustering of significant genes was conducted using *r*-log-transformed values. Gene enrichment and functional annotation analysis for the significant gene list were performed on the basis of gene set enrichment analysis (GSEA; www.gsea-msigdb.org/gsea/msigdb/mouse/annotate.jsp) and the Kyoto Encyclopedia of Genes and Genomes (KEGG) pathway database (https://www.genome.jp/kegg/), respectively. All data analysis and visualization of differentially expressed genes were conducted using R 4.2.2 (www.r-project.org).

### Deconvolution of bulk RNA-seq data

Cell-type composition and cell-type-specific gene expression were jointly estimated from mouse liver bulk RNA-seq data using BayesPrism (v.2.2.2)^40^, with gene-level raw read counts as input. The mouse liver sc/snRNA-seq atlas (GSE192742)^41^ was used as a single-cell reference. Myeloid subtypes were grouped into three lineage-based macrophage cell types: resident macrophages, monocyte-derived macrophages and monocyte states. Dendritic subsets were collapsed into a single dendritic cell type, and neutrophils and basophils were grouped into a granulocyte cell type. A complete cell state-to-type mapping is provided in Supplementary Data 1. Reference genes were filtered using cleanup.genes (species = “mm”; gene.group = c(“Rb”, “Mrp”, “other_Rb”, “chrM”, “chrX”, “chrY”); exp.cells = 5). Genes were further restricted to protein-coding genes using org.Mm.eg.db (v.3.18.0)^42^. Deconvolution was performed with key = NULL. Estimation reliability was evaluated using the posterior coefficient of variation of the inferred cell-type proportions, as implemented in BayesPrism. Cell-type-specific *Gadd45b* expression was extracted from the BayesPrism posterior using get.exp (state.or.type = “type”).

### Isolation and culture of primary hepatocytes, hepatic stellate cells and KCs from the mouse liver

Hepatocytes and nonparenchymal cells (NPCs) were isolated from 8-week-old male C57BL/6 mice by collagenase perfusion method^43^. Primary hepatocytes were separated from NPCs by centrifugation at 500 rpm for 4 min and further purified by 50% Percoll density gradient centrifugation at 1250 rpm for 5 min. The NPC fraction was resuspended in 20% Opti-prep and subjected to 11.5% Opti-prep density gradient centrifugation at 3000 rpm for 17 min to separate and enrich hepatic stellate cells (HSCs) and Kupffer cells (KCs). Cells were plated in Primaria six-well plates (353846, Corning) with medium 199 (M4530, Sigma-Aldrich) supplemented with 10% fetal bovine serum (FBS), 1% antibiotics and 10 nM dexamethasone.

After attachment, primary hepatocytes or KCs were infected with adenoviruses for 48 or 72 h. Primary hepatocytes were maintained in medium 199 without 10% FBS and were treated with bovine serum albumin (BSA)-conjugated palmitic acid (PA; P9767, Sigma-Aldrich), 10 ng/ml recombinant mouse tumor necrosis factor-α (TNFα; 410-MT-010, R&D Systems), 5 ng/ml recombinant mouse transforming growth factor-β1 (TGFβ1; 7666−ΜΒ−005, R&D Systems) or 100 μM pyrrolidine dithiocarbamate (PDTC; ab141406, Abcam). KCs were treated with 50 ng/ml recombinant mouse TNFα.

### Cell culture and transfection

RAW 264.7 (TIB-71, ATCC, VA), LX-2 (SCC064, Merck Millipore) and Huh7 (JCRB0403, JCRB Cell Bank) cells were cultured in Dulbecco’s modified Eagle medium (SH30243.01, HyClone) supplemented with FBS (10% for RAW 264.7 and Huh7, 2% for LX-2 cells), 1% antibiotics and 1% L-glutamine (for RAW 264.7 and LX-2 cells) and maintained in a humidified atmosphere of 5% CO_2_ at 37 °C. RAW 264.7 cells were transfected using Lipofectamine LTX plus reagent system (15338100, Invitrogen), while LX-2 cells and Huh7 cells were transfected using Lipofectamine 3000 (L3000015, Invitrogen), according to the manufacturer’s instructions.

### Plasmids and recombinant adenoviruses

Mouse and human *Gadd45b* were inserted into the pcDNA3-Flag expression vector. Adenoviruses expressing GFP control, *Gadd45b*, unscrambled nonspecific RNAi control (US) and sh*Gadd45b* were used as previously described^44^. For animal experiments, the adenoviruses were purified on a CsCl gradient, dialyzed against PBS buffer containing 10% glycerol, and stored at −80 °C.

### Preparation of conditioned medium (CM) and treatment

Forty-eight hours after transfection of RAW 264.7 cells or LX-2 cells, cell supernatants were collected and centrifuged to remove cell debris, and the resulting CM was collected. Subsequently, mouse primary hepatocytes, mouse primary HSCs or LX-2 cells were incubated with the CM for 24 or 48 h. At the time of CM collection, RAW 264.7 cells were collected for mRNA expression analysis.

### Western blot analysis

Mouse liver lysates were separated by SDS–PAGE and transferred onto polyvinylidene fluoride membranes. The membranes were incubated with the primary antibodies (anti-HSP90 at 1:5,000, others at 1:1,000 dilution), followed by HRP-conjugated secondary antibody and were visualized using an enhanced chemiluminescence detection (GE Healthcare). The antibodies involved in this study are presented in Supplementary Table 2.

### Huh7 spheroid formation

A total of 3 × 10^3^ viable Huh7 cells were seeded in 100 µl of cell culture medium per well of U-shaped ultralow-attachment 96-well plates (7007, Corning). After seeding, the plate was centrifuged at 50*g* for 5 min, and cells were allowed to aggregate and form spheroids (1 spheroid per well) at 37 °C for 72 h. The medium was replaced with macrophage-derived CM every 48 h for the following 9–10 days. For PDTC experiments, Huh7 spheroids were treated with 100 μM PDTC, which was replenished together with CM every 48 h. Brightfield images of the spheroids were taken at specific time points using a Research Inverted Microscope (IX73, Olympus). The overall volume of the spheroid was determined using ImageJ software.

### Statistical analysis

Detailed information about the value of *n*, along with significance and error bars, is provided in the legends of each figure. Statistical differences between two experimental groups were evaluated by the two-tailed unpaired Student’s *t*-test, and one-way analysis of variance (ANOVA) tests were used for multiple group comparison tests by using GraphPad Prism 8.0.1 (GraphPad Software). Data are shown as mean ± s.d. or mean ± s.e.m. The threshold for statistical significance was set at *P* values less than 0.05; *P < 0.05, **P < 0.01, ***P < 0.001.

## Results

### MASH-associated metabolic stress drives hepatocarcinogenesis under controlled fibrotic stress in C57BL/6N mice

**Fig. 1:**
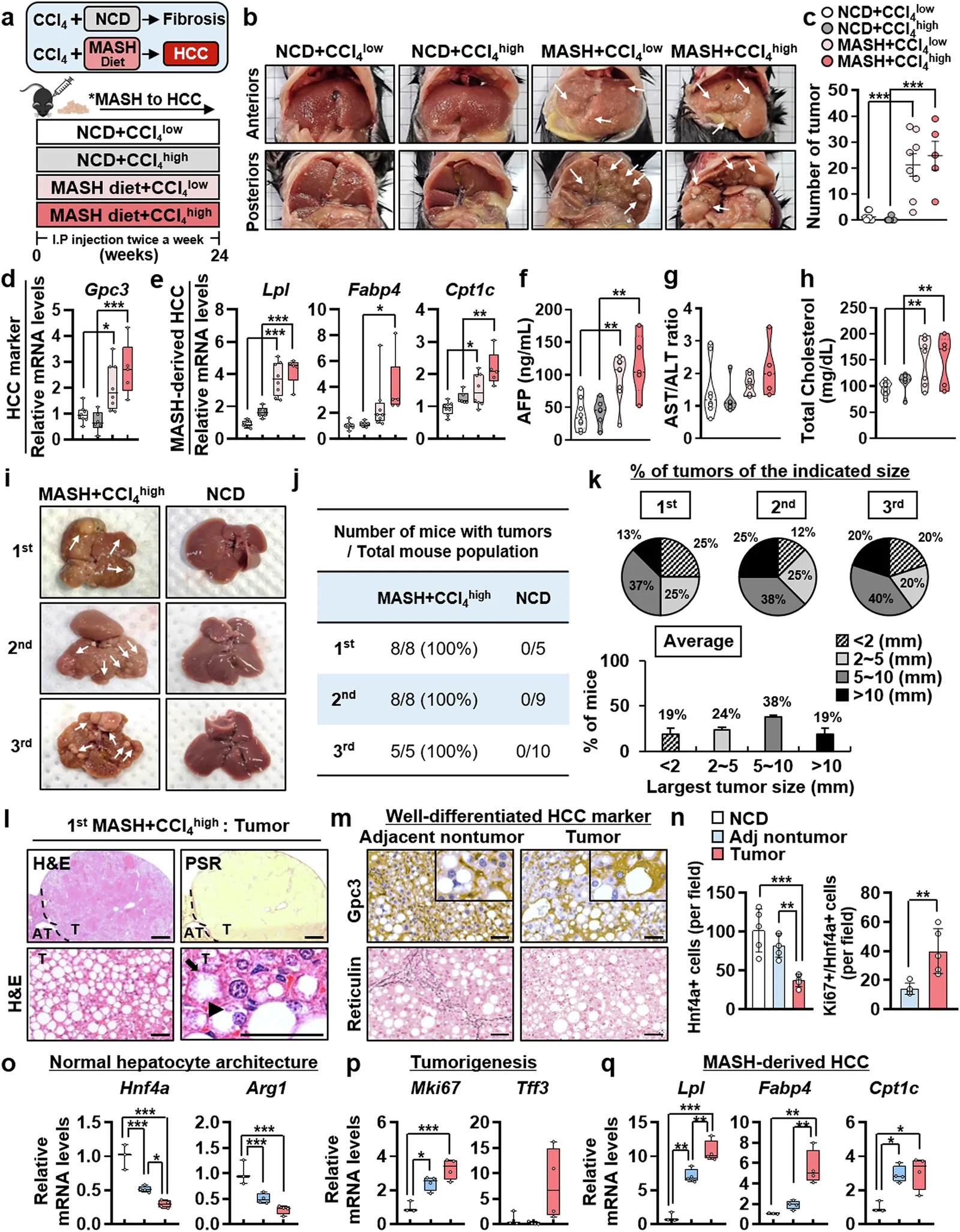
MASH-provoking diet promotes reproducibly well-differentiated HCC in C57BL/6N mice. **a** Experimental design for the establishment of a MASH-associated HCC mouse model: Groups include NCD + CCl_4_^low^ (*n* = 8), NCD + CCl_4_^high^ (*n* = 6), MASH diet + CCl_4_^low^ (*n* = 8) and MASH diet + CCl_4_^high^ (*n* = 5). **b** Representative gross appearance of mouse livers, with white arrows indicating surface tumor nodules. **c** The number of visible tumors ≥1.0 mm per mouse at 24 weeks. **d**, **e** Reverse transcription (RT)–qPCR analysis of an HCC marker (**d**) and MASH-derived HCC markers (**e**) in mouse liver tumors. The box plots show the distribution of data based on its five-number summary (minimum, lower quartile, median, upper quartile and maximum). *P < 0.05, **P < 0.01, ***P < 0.001, analyzed by one-way ANOVA followed by Tukey’s test. **f**–**h** Violin plots of serum AFP (**f**) AST per ALT ratio (**g**) and total cholesterol levels (**h**) at 24 weeks. The thick dashed line and thin dotted line represent the median and interquartile range. **P < 0.01, analyzed by one-way ANOVA. **i**–**n** The MASH-associated HCC mouse model was generated in three independent experiments to validate reproducibility. 1^st^, the first experiment (NCD, *n* = 5; MASH diet + CCl_4_^high^, *n* = 8); 2^nd^, the second experiment (NCD, *n* = 9; MASH diet + CCl_4_^high^, *n* = 8); 3^rd^, the third experiment (NCD, *n* = 10; MASH diet + CCl_4_^high^, *n* = 5). **i** Representative gross appearance of mouse livers, with white arrows indicating surface tumor nodules. **j** Table showing the number of mice with tumors in the NCD or MASH diet + CCl_4_^high^ group. **k** Percentages of mice with the indicated maximum tumor sizes in three independent experiments (top) and the overall average percentage (bottom). **l** Representative H&E and PSR staining of liver tumor sections from MASH diet + CCl_4_^high^ mice (20×; scale bar, 1 mm), shown from the first of three independent experiments. In the H&E sections, hepatocyte ballooning (arrow) and Mallory–Denk bodies (arrowhead) are visible (400×; scale bar, 50 μm). **m** Representative Gpc3 and Reticulin (for fiber visualization) staining of liver tumor sections from MASH diet + CCl_4_^high^ mice (400×; scale bar, 50 μm), shown from the first of three independent experiments. The inset in the Gpc3 panel clearly shows the hepatocyte nucleus and cytoplasm. **n** The quantification of Hnf4a-positive cells and Ki67-positive cells among Hnf4a-positive cells in liver tissues from NCD, adjacent nontumor and tumor sections of MASH diet + CCl_4_^high^ mice (*n* = 5 per group). **o**–**q** RT–qPCR analysis of normal hepatocyte architecture markers (**o**), tumorigenesis markers (**p**) and MASH-derived HCC markers (**q**) in liver tissues from NCD, adjacent nontumor and tumor sections of MASH diet + CCl_4_^high^ mice (NCD, *n* = 3; MASH diet + CCl_4_^high^, *n* = 4). The box plots show data distribution. *P < 0.05, **P < 0.01, ***P < 0.001, analyzed by one-way ANOVA. Data in **c** are presented as mean ± s.e.m., and data in **n** are presented as mean ± s.d. **P < 0.01, ***P < 0.001, analyzed by one-way ANOVA followed by Tukey’s test.

To investigate the effect of MASH-associated metabolic stress as a primary driver of liver tumorigenesis, C57BL/6N mice were fed a MASH-inducing diet, in comparison with normal chow, under controlled CCl_4_-induced fibrotic stress (Fig. 1a, b). All mice receiving the MASH-provoking diet developed multiple liver tumors, accompanied by significant increases in liver weight and liver-to-body weight ratio, reflecting liver lipid accumulation and inflammatory remodeling (Fig. 1c and Supplementary Fig. 1a). These tumors exhibited robust expression of the HCC marker *Gpc3*^45^, the proliferation marker *Mki67*, and hepatic progenitor cell markers *Krt19*, *Krt7*, *Epcam* and *Ncam1*^46,47^ (Fig. 1d and Supplementary Fig. 1b,c), as well as upregulation of the Lpl–Fabp4–Cpt1c metabolic axis^48^, a key signature of MASH-derived HCC (Fig. 1e). Furthermore, they exhibited elevated expression of genes involved in fatty acid (FA) uptake, inflammation and fibrosis, while lipogenic genes remained unchanged, suggesting that increased intracellular FAs may mediate inflammation progression at this lesion stage (Supplementary Fig. 1d–g). In parallel, these lesions were associated with elevated circulating AFP, AST/ ALT ratio and total cholesterol (Fig. 1f–h). These results demonstrate that progressive inflammation and metabolic dysfunction in MASH act as critical drivers of hepatocarcinogenesis.

To establish a robust preclinical platform for mechanistic studies and therapeutic evaluation of the MASH-to-HCC transition, we developed a mouse model that reproducibly formed tumors in all mice across three independent experiments. Tumor size distribution was as follows: 19% >10 mm, 38% between 5 mm and 10 mm, 24% between 2 mm and 5 mm, and 19% <2 mm (Fig. 1i–k). Across all experiments, tumors were well demarcated from the surrounding fibrotic liver tissue and exhibited reduced collagen deposition compared with the adjacent fibrotic areas, as shown by H&E and PSR staining (Fig. 1l and Supplementary Fig. 2a,b). Tumor tissues displayed hallmark features of steatohepatitic HCC, including extensive lipid accumulation covering more than two-thirds of the tissue area, hepatocyte ballooning and Mallory–Denk bodies (Fig. 1l and Supplementary Fig. 2a,b). Inflammatory loci, as assessed by F4/80 staining, were more prominent in adjacent nontumor tissues, whereas hepatic crown-like structures^49^—formed by macrophages surrounding lipid droplets and dead cells—were enriched within tumor tissues (Supplementary Fig. 2c). These macrophage-associated pathological features are consistent with MASH-driven tumorigenesis.

Gpc3 and reticulin staining serve as critical markers for distinguishing well-differentiated HCC from hepatocellular adenoma (HCA) and dysplastic liver nodules^45,50–52^. Increased cytoplasmic Gpc3 expression in hepatocytes and loss of reticulin fibers in tumor regions compared with adjacent nontumor tissue classified the tumor as well-differentiated HCC^50,53^ (Fig. 1m and Supplementary Fig. 2d,e). Ck19 immunohistochemistry is a useful tool for assessing liver architecture and detecting ductular reaction^54^, which aids in distinguishing true stromal invasion by tumor cells from pseudo-infiltration caused by peritumoral inflammation in HCC^55,56^. Ductular reaction was frequently observed in adjacent nontumor tissue but was largely absent in tumor tissue (Supplementary Fig. 2f). Overall, histological changes are summarized in Supplementary Fig. 3 and Supplementary Table 3.

Consistent with these histological findings, the hepatocyte marker Hnf4a was downregulated in tumor tissue compared with normal chow-fed liver tissues and adjacent nontumor tissues, whereas Ki67 positivity was increased among Hnf4a-positive cells, indicating elevated hepatocyte proliferation within tumors (Fig. 1n and Supplementary Fig. 2g,h). At the molecular level, *Hnf4a* and *Arg1* were downregulated, whereas *Tff3*^57,58^ and *Mki67* were upregulated (Fig. 1o, p). The Lpl–Fabp4–Cpt1c metabolic axis was also elevated (Fig. 1q). Consistent with these findings, a similar expression pattern was observed in the STAM mouse model dataset, a well-established model of MASH-derived HCC (Supplementary Fig. 4). These histological and molecular features demonstrate that tumors arising in this model represent well-differentiated, MASH-derived HCC.

### Gadd45b is identified as a proinflammatory factor in diet-induced MASH-to-HCC progression

**Fig. 2:**
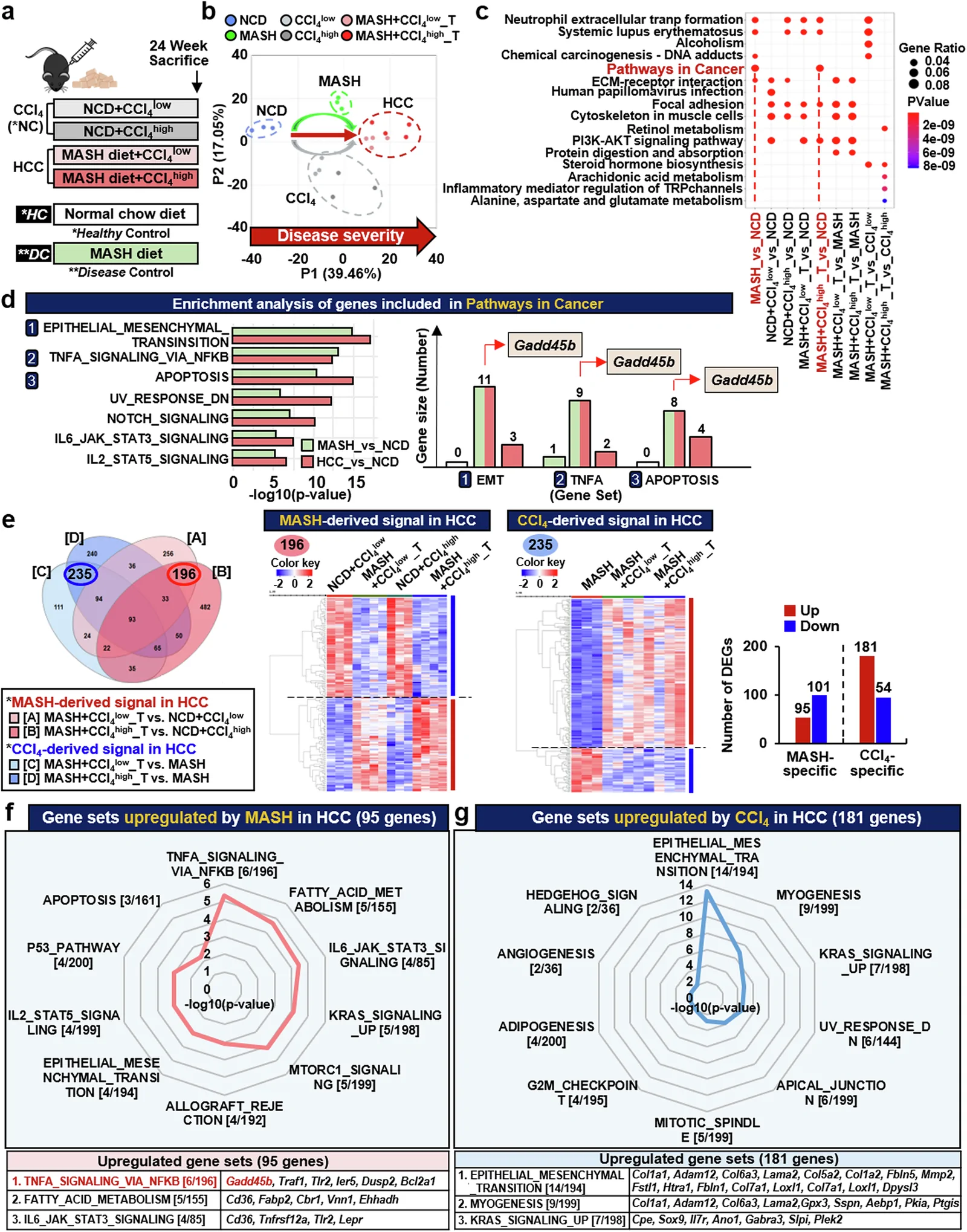
Informatics-based identification of Gadd45b linking diet-driven MASH to HCC. **a** For liver RNA-seq profiling, the groups include the NCD + CCl_4_^low^ (*n* = 3), NCD + CCl_4_^high^ (*n* = 3), MASH diet + CCl_4_^low^ (tumor tissue, *n* = 4), MASH diet + CCl_4_^high^ (tumor tissue, *n* = 4), NCD (*n* = 3) and MASH diet (*n* = 3). **b** PCA plots of RNA-seq data. **c** KEGG pathway enrichment analysis of differentially expressed genes (DEGs) in each comparison group. **d** Bar charts showing Hallmark gene set enrichment of upregulated genes involved in KEGG Pathways in Cancer, along with the number of overlapping or uniquely represented genes. **e** Venn diagram showing 196 DEGs associated with MASH in HCC and 235 DEGs associated with CCl_4_ in HCC, with a heatmap of their expression and a bar graph indicating the number of upregulated and downregulated DEGs. **f**, **g** Radial line graphs showing Hallmark pathways upregulated in HCC by MASH (**f**) or CCl_4_ (**g**), with associated gene lists. Bracketed values represent overlap/input gene numbers.

Because HCC developed selectively in the MASH diet plus CCl_4_ group, but not in the NCD plus CCl_4_ group, we compared their liver transcriptomes across all four groups (NCD, MASH, NCD+CCl_4_ and MASH+CCl_4_) with particular focus on distinguishing the HCC-bearing MASH+CCl_4_ group from the NCD+CCl_4_ group, to identify MASH-specific tumorigenic transcriptional programs (Fig. 2a). Principal component analysis (PCA) plots of mRNA-seq data revealed clear separation among the groups (Fig. 2b). KEGG pathway enrichment analysis identified ‘Pathways in Cancer’ as significantly enriched in both the MASH and HCC (MASH+CCl_4_^high^_T) groups (Fig. 2c). To further dissect the transcriptional programs underlying this enrichment, hallmark pathway enrichment analysis was performed for each group (Fig. 2d). Three key gene sets— EPITHELIAL MESENCHYMAL_ TRANSITION, TNFA_SIGNALING_VIA_ NF-KB, and APOPTOSIS—were commonly and significantly upregulated (Fig. 2d and Supplementary Fig. 5a,b). Notably, *Gadd45b* was consistently enriched across all three gene sets, suggesting its potential role in MASH-driven tumorigenesis (Fig. 2d and Supplementary Fig. 5c).

To identify MASH-specific signals in HCC, 196 genes differentially expressed in the MASH+CCl_4__T (HCC) group compared to the NCD+CCl_4_ group were analyzed and visualized in a heatmap (Fig. 2e). Hallmark GSEA of the 95 upregulated genes identified TNFA_SIGNALING_VIA_NF-KB and FATTY_ACID_METABOLISM as the top enriched pathways (Fig. 2f). These pathways included *Gadd45b* and the FA transporters *Cd36* and *Fabp2*, highlighting a potential role for Gadd45b linking FA metabolism to liver inflammation (Fig. 2f). By contrast, CCl_4_-specific signals in HCC were enriched for EPITHELIAL_MESENCHYMAL_TRANSITION, indicating mechanistic divergence from MASH-driven pathways (Fig. 2g). qPCR validation confirmed that *Gadd45b* and proinflammatory TNFα–NF-kB target genes were significantly upregulated in the MASH and HCC groups, whereas the NCD+CCl_4_ group showed a trend of increased *Gadd45b* and *Tgfb1* expression, but these differences were not statistically significant. This supported the finding that MASH-associated inflammation underlies tumorigenesis and is recapitulated in RNA-seq (Supplementary Fig. 5d,e).

**Fig. 3:**
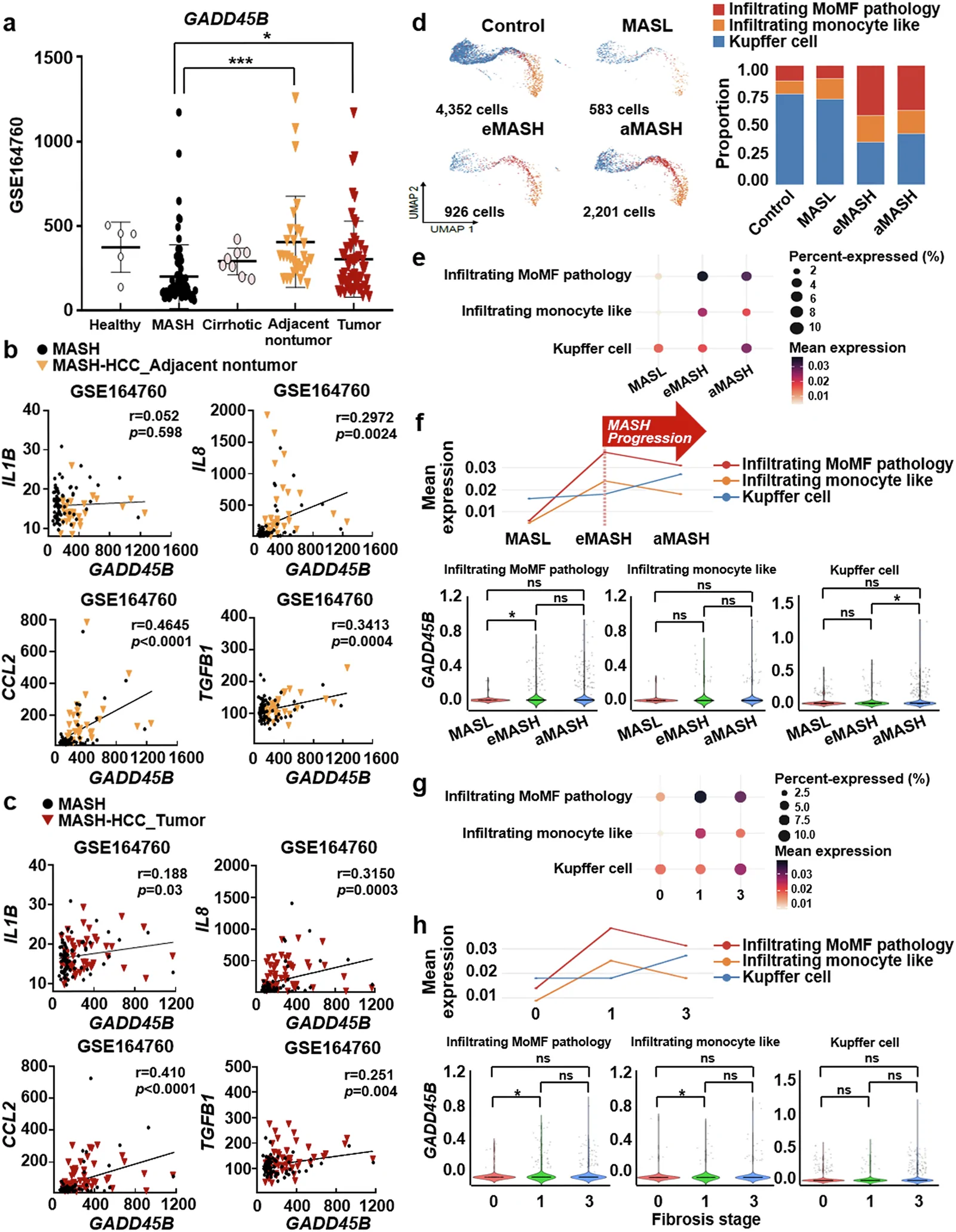
Macrophage-associated Gadd45b tracks inflammatory activation during human MASH-to-HCC progression. **a**–**c** *GADD45B* expression and its correlation with the expression levels of inflammatory factors were analyzed based on the GEO dataset (GSE164760). **a** *GADD45B* expression levels in patients with MASH and cirrhosis and in adjacent nontumor and tumor from patients with MASH–HCC. **b**, **c** Correlation of *GADD45B* expression with the expression levels of *IL1B*, *IL8*, *CCL2* and *TGFB1* in MASH and MASH–HCC adjacent nontumor (**b**), and MASH and MASH–HCC tumor (**c**). Pearson’s correlation coefficients (*r*) and *P* values are represented for each analysis. **d**–**h** Single-cell transcriptomic analysis showing disease stage-dependent remodeling of macrophage populations and *GADD45B* expression based on the GEO dataset (GSE289173). **d** Clustering of major macrophage cell types. Uniform manifold approximation and projection (UMAP) plot showing major macrophage types (left) and their proportions by disease stage (right). **e** Dot plot showing disease stage-dependent changes in macrophage subtype proportions. **f** Line graph and violin plot showing changes in *GADD45B* expression across disease stages and macrophage cell type. *P < 0.05, analyzed by one-way ANOVA followed by Tukey’s test. **g** Dot plot showing fibrosis stage-dependent changes in macrophage subtype proportions. **h** Line graph and violin plot showing changes in *GADD45B* expression across fibrosis stages and macrophage cell type. *P < 0.05, analyzed by one-way ANOVA followed by Tukey’s test.

To determine whether Gadd45b identified as a target linking diet-driven MASH to HCC in a mouse model is similarly relevant in human disease, we analyzed public human gene expression datasets. Bulk RNA-seq analysis of human liver samples^59^ revealed a progressive increase in *GADD45B* expression during MASH-to-HCC progression, which was positively correlated with the expression of proinflammatory TNFα–NF-kB target genes, including *IL1B*, *CCL2* and *TGFB1* (Fig. 3a–c). Single-cell transcriptomic analysis^60^ further revealed disease stage-dependent remodeling of macrophage populations, accompanied by elevated *GADD45B* expression across macrophage subsets (Fig. 3d–h). Specifically, *GADD45B* expression surged in newly recruited infiltrating macrophages, including lipid-associated macrophages, at early stages of MASH and further increased in liver-resident KCs at advanced stages (Fig. 3e, f). This stage-dependent pattern of *GADD45B* expression was similarly observed across macrophage subsets during fibrosis progression (Fig. 3g, h). These findings support a potential role for Gadd45b in linking chronic inflammation to protumorigenic programs.

Consistent with these findings, BayesPrism deconvolution^40^ of mouse liver bulk RNA-seq data using a mouse single-cell/single-nucleus RNA-seq reference dataset (GSE192742)^41^ revealed expansion of monocyte-derived macrophages and reduction of resident KCs in MASH, followed by partial restoration of KCs during HCC progression (Supplementary Fig. 6a). Moreover, *Gadd45b* expression tracked with disease stage-dependent remodeling of macrophage subsets, supporting a conserved role for Gadd45b in macrophage dynamics during MASH-to-HCC progression (Supplementary Fig. 6b). Detailed cell type- and cell state-level deconvolution results are provided in Supplementary Data 1.

### Gadd45b acts as a macrophage-driven inflammatory early responder during the transition from steatosis to steatohepatitis

**Fig. 4:**
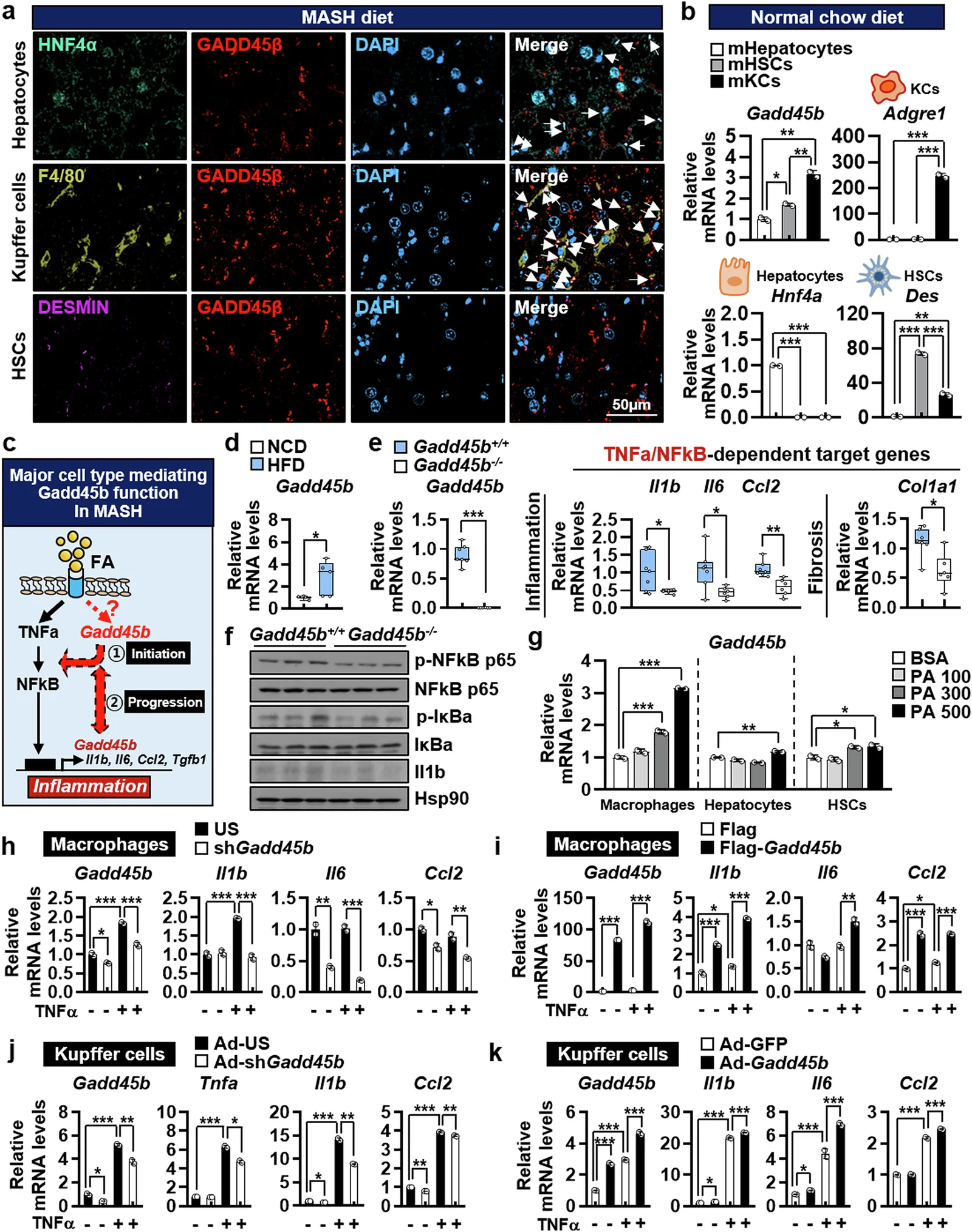
Gadd45b regulates macrophage-intrinsic inflammatory responses under MASH conditions. **a** Immunofluorescence staining of liver sections from the MASH group. Representative images showing co-localization of HNF4α (cyan), F4/80 (yellow), DESMIN (magenta) and Gadd45β (red). Nuclei were counterstained with DAPI (blue) (200×; scale bar, 50 μm). **b** RT–qPCR analysis of mRNA levels of *Gadd45b*, *Adgre1*, *Hnf4a* and *Des* in primary hepatocytes, HSCs and KCs from C57BL/6N mice. **c** Schematic diagram showing the hypothesized role of Gadd45b in FA-induced proinflammatory responses via the TNFα–NF-kB signaling pathway under MASH conditions. **d** RT–qPCR analysis of *Gadd45b* mRNA level in the livers of mice fed either an NCD or HFD for 12 weeks (*n* = 5 per group). **e**, **f** Nine-week-old *Gadd45b*^+/+^ mice (*n* = 7) and *Gadd45b*^−/−^ mice (*n* = 6) were fed an HFD for 10 weeks. **e** RT–qPCR analysis of mRNA levels of *Gadd45b* and TNFα–NF-kB target genes. **f** Western blot analysis showing the effects of *Gadd45b* knockout on phospho- or total protein levels of NF-kB p65, IkBa and Il1b in mouse livers. **g** RT–qPCR analysis of *Gadd45b* mRNA level in RAW 264.7 cells treated with BSA or 100, 300 or 500 μM PA for 24 h, and in primary hepatocytes and LX-2 cells treated with BSA or PA for 12 h. **h**, **i** RT–qPCR analysis of inflammatory gene expression in RAW 264.7 cells following *Gadd45b* knockdown (**h**) or overexpression (**i**). Cells transfected with (**h**) the unscrambled nonspecific RNAi control vector (US), U6-sh*Gadd45b*, or (**i**) pcDNA3-flag or pcDNA3-flag-*Gadd45b* were treated with or without 50 ng/ml TNFα for 2 h. **j**, **k** RT–qPCR analysis of inflammatory gene expression in primary KCs following *Gadd45b* knockdown (**j**) or overexpression (**k**). Cells infected with either (**j**) Ad-US control, Ad-sh*Gadd45b* or (**k**) Ad-GFP or Ad-*Gadd45b* were treated with or without 50 ng/ml TNFα for 2 h. Data in **b** and **g**–**k** are presented as mean ± s.d., analyzed by one-way ANOVA followed by Tukey’s test; data in **d** and **e** are shown as box plots representing the data distribution, analyzed by *t*-test. *P < 0.05, **P < 0.01, ***P < 0.001.

Despite the established link between lipid accumulation and inflammation in MASH, our data indicate a more stage- and cell-type-dependent pattern of Gadd45b expression. Notably, *GADD45B* expression was reduced during simple steatosis but reinduced during inflammatory stages in human patients. This reactivation coincided with a marked increase in *GADD45B* expression in hepatic macrophages in humans. Therefore, to identify the major liver cell types expressing Gadd45b under MASH conditions in a mouse model, we performed immunohistochemistry on liver tissues from mice fed MASH-inducing diets, using antibodies against Gadd45b together with specific markers for KCs, hepatocytes and HSCs (Fig. 4a). Under MASH conditions, Gadd45β predominantly colocalized with the macrophage marker F4/80, moderately with the hepatocyte marker HNF4α, and rarely with the HSC marker DESMIN (Fig. 4a). In line with this, in healthy livers, Gadd45b was primarily expressed in KCs (Fig. 4b and Supplementary Fig. 7). These findings suggest that, under MASH conditions, Gadd45b functions as an early inflammatory responder in macrophages, rather than as a direct driver of lipid accumulation.

FAs are key inflammatory mediators linking MASH and HCC^61,62^ As shown in Fig. 2f, transcriptomic profiling identified Gadd45b as a candidate gene potentially bridging FA metabolism and inflammation during MASH-to-HCC progression. However, it is unclear which cell types of the liver Gadd45b is induced under FA-dependent inflammatory conditions and how it functionally contributes to MASH pathogenesis (Fig. 4c). Therefore, we examined the expression and function of Gadd45b in the liver under FA-induced inflammatory stress following 12 weeks of HFD feeding (Fig. 4d–f and Supplementary Fig. 8a,b). *Gadd45b* expression was significantly increased, concomitant with upregulation of proinflammatory TNFα–NF-kB target genes (Fig. 4d and Supplementary Fig. 8a). Depletion of *Gadd45b* suppressed the expression of these inflammatory factors at both the mRNA and protein levels under the same conditions (Fig. 4e, f and Supplementary Fig. 8b).

To determine whether FA exposure directly induces Gadd45b in specific liver cell types, PA, the most abundant saturated FA, was applied to macrophages (RAW 264.7 cells), primary hepatocytes and HSCs (LX-2 cells), resulting in markedly stronger induction in macrophages (Fig. 4g). This induction was accompanied by increased expression of *Tnfa* and its downstream target genes, consistent with the involvement of TNFα signaling in Gadd45b regulation under MASH conditions (Supplementary Fig. 9a). Short-term treatment with TNFα for 2 h notably increased *Gadd45b* expression in both RAW 264.7 macrophages and KCs, concomitant with induction of proinflammatory genes, indicating that Gadd45b is rapidly induced in response to early proinflammatory signals (Supplementary Fig. 9b–e). Functionally, knockdown of *Gadd45b* suppressed both basal and TNFα–NF-κB-induced proinflammatory gene expression in both RAW 264.7 macrophages and primary KCs, whereas ectopic *Gadd45b* expression enhanced their expression (Fig. 4h–k). By contrast, although TNFα induced *Gadd45b* expression in primary hepatocytes, its depletion did not affect proinflammatory gene expression, indicating a limited functional role of hepatocyte Gadd45b in early MASH-associated inflammation (Supplementary Fig. 9f–h). These data indicate that Gadd45b functions as both a downstream responder to inflammatory stimuli and an upstream amplifier of macrophage-mediated inflammation under MASH conditions.

### Macrophage Gadd45b amplifies liver inflammation through a feed-forward Gadd45b–TNFα–NF-kB axis in hepatocytes

**Fig. 5:**
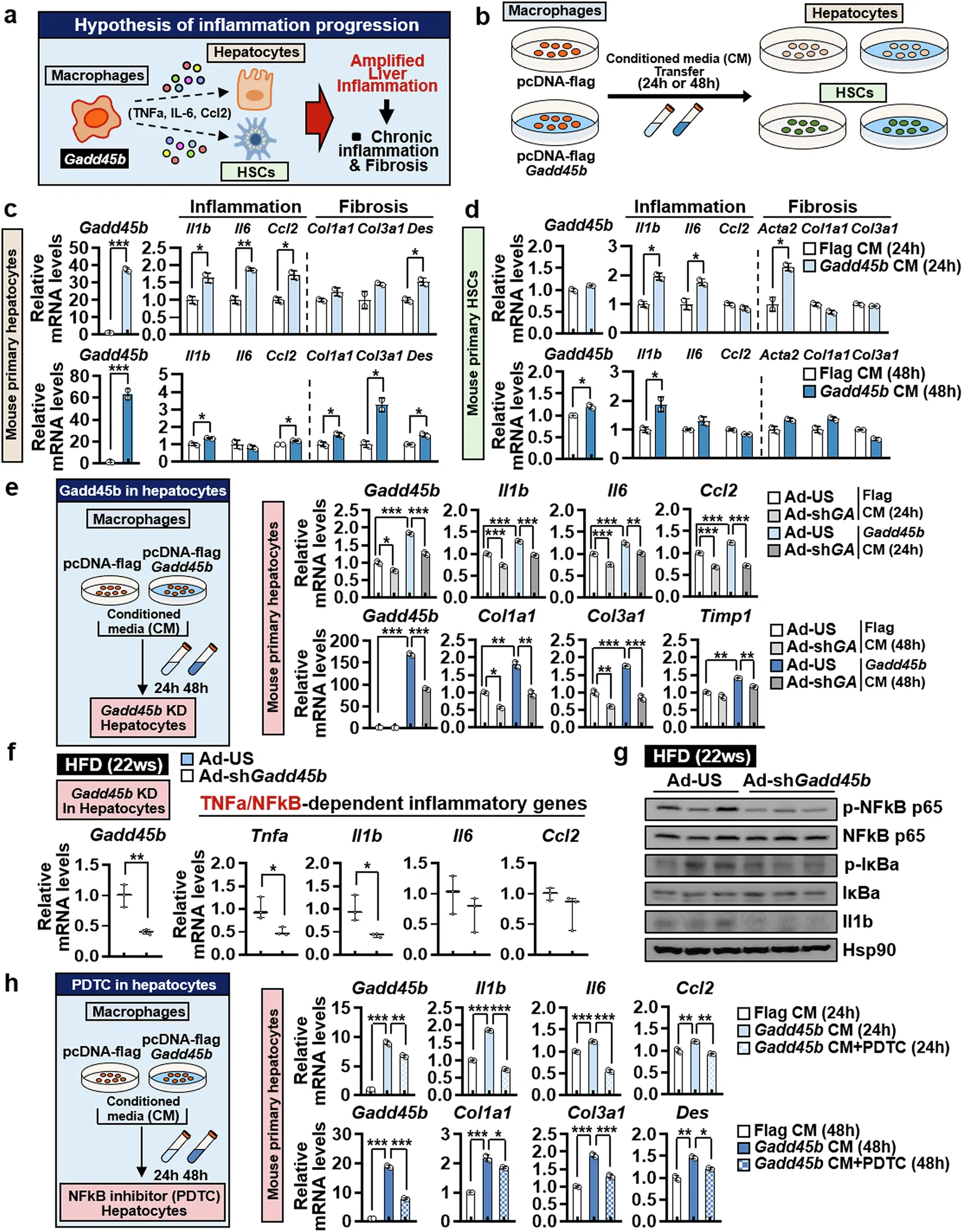
Hepatocyte Gadd45b is required for macrophage-derived inflammatory signal amplification. **a** Schematic diagram showing the proposed role of macrophage Gadd45b in amplifying the proinflammatory response by promoting inflammation in neighboring hepatic cells. **b** Schematic diagram of the experimental design. CM was collected from the supernatants of macrophages transfected with either pcDNA3-flag or pcDNA3-flag-*Gadd45b*. Mouse primary hepatocytes or HSCs were subsequently cultured with the CM for 24 or 48 h. **c**, **d** RT–qPCR analysis showing the effects of CM derived from RAW 264.7 cells on the expression of inflammatory and fibrotic markers in mouse primary hepatocytes (**c**) or primary HSCs (**d**). **e** RT–qPCR analysis of the effects of *Gadd45b* knockdown on the mRNA levels of inflammatory or fibrotic markers in mouse primary hepatocytes treated with RAW 264.7 cell-derived CM for 24 or 48 h. Primary hepatocytes were infected with either Ad-US control or Ad-sh*Gadd45b* before CM treatment. **f**, **g** Eight-week-old C57BL/6 male mice were fed an HFD for 22 weeks and then infected with either Ad-US control or Ad-sh*Gadd45b* for 7 days (*n* = 3 per group). RT–qPCR analysis (**f**) of mRNA levels of inflammatory genes in mouse livers. The box plot shows data distribution. *P < 0.05, **P < 0.01, analyzed by *t*-test. Western blot analysis (**g**) showing the effects of *Gadd45b* knockdown on phospho- or total protein levels of NF-kB p65, IkBa and Il1b in mouse livers. **h** RT–qPCR analysis of the effects of PDTC on inflammatory and fibrotic marker expression in mouse primary hepatocytes exposed to RAW 264.7 cell-derived CM for 24 or 48 h, with 100 μM PDTC pretreatment for 2 h followed by continuous exposure during CM treatment. Data in **c** and **d** are presented as mean ± s.d. and analyzed by *t*-test; data in **e** and **h** are presented as mean ± s.d. and analyzed by one-way ANOVA followed by Tukey’s test. *P < 0.05, **P < 0.01, ***P < 0.001.

Given that Gadd45b functions as both an NF-kB effector and a metabolic stress sensor, we hypothesized that proinflammatory factors secreted by *Gadd45b*-expressing macrophages induce *Gadd45b* expression and an inflammatory response in neighboring hepatic cells, thereby amplifying hepatic inflammation. To test this hypothesis, hepatocytes and HSCs were treated with CM collected from *Gadd45b*-expressing macrophages for 24 h or 48 h (Fig. 5a, b). At the time of CM collection, *Gadd45b*-overexpressing macrophages exhibited increased expression of *Gadd45b* and inflammatory genes, confirming the establishment of a proinflammatory macrophage state (Supplementary Fig. 10a,b). In primary hepatocytes, CM treatment for 24 h significantly increased the expression of *Gadd45b* and inflammatory genes, whereas prolonged exposure for 48 h induced fibrogenic genes expression, consistent with sustained inflammatory signaling driving a profibrogenic transcriptional program (Fig. 5c and Supplementary Fig. 10a). By contrast, CM treatment elicited only a weak induction of *Gadd45b* in mouse primary HSCs at 48 h (Fig. 5d and Supplementary Fig. 10b) and failed to induce *Gadd45b* expression in LX-2 cells (Supplementary Fig. 10c). Unlike hepatocytes, these limited changes in *Gadd45b* expression were not accompanied by substantial changes in inflammatory or fibrogenic gene expression (Fig. 5d and Supplementary Fig. 10c). Furthermore, CM derived from *Gadd45b*-expressing HSCs induced *Gadd45b* and fibrogenic gene expression in hepatocytes at 48 h, but not at 24 h, further supporting hepatocytes as the primary cellular targets and amplifiers of Gadd45b-driven chronic inflammatory signaling in the liver (Supplementary Fig. 11a).

To determine whether hepatocyte Gadd45b mediates inflammatory signaling triggered by *Gadd45b*-expressing macrophages, we knocked down *Gadd45b* in hepatocytes and treated them with macrophage CM for 24 or 48 h (Fig. 5e). Knockdown of *Gadd45b* markedly attenuated the CM-induce expression of inflammatory and fibrogenic genes in hepatocytes, highlighting a pivotal role for hepatocyte Gadd45b in mediating macrophage-driven paracrine inflammatory signaling (Fig. 5e and Supplementary Fig. 11b). To further assess the in vivo relevance of these finding, we performed hepatocyte-specific *Gadd45b* knockdown using adenoviral vectors in mice subjected to a 22-week HFD. Depletion of hepatocyte *Gadd45b* substantially suppressed chronic HFD-induced activation of the TNFα–NF-kB pathway, as indicated by reduced expression of proinflammatory factors at both the mRNA and protein levels (Fig. 5f, g and Supplementary Fig. 12). To further determine whether macrophage-derived inflammatory signaling is mediated through a TNFα–NF-κB feed-forward loop in hepatocytes, hepatocytes were pretreated with PDTC, a pharmacological inhibitor of NF-κB activation, before stimulation with macrophage CM (Fig. 5h). PDTC significantly suppressed CM-induced expression of inflammatory and fibrogenic genes, recapitulating the effects observed following *Gadd45b* knockdown in hepatocytes (Fig. 5h). These findings suggest that hepatocyte Gadd45b promotes macrophage-induced inflammatory signaling, at least in part, through activation of a TNFα–NF-κB feed-forward loop. Collectively, these findings identify hepatocyte Gadd45b as a key mediator of macrophage-driven inflammatory signaling by amplifying the TNFα–NF-kB feed-forward loop under chronic inflammatory conditions such as MASH.

### Macrophage and hepatocyte Gadd45b promote inflammatory signaling linked to partial EMT-like programs and spheroid formation

**Fig. 6:**
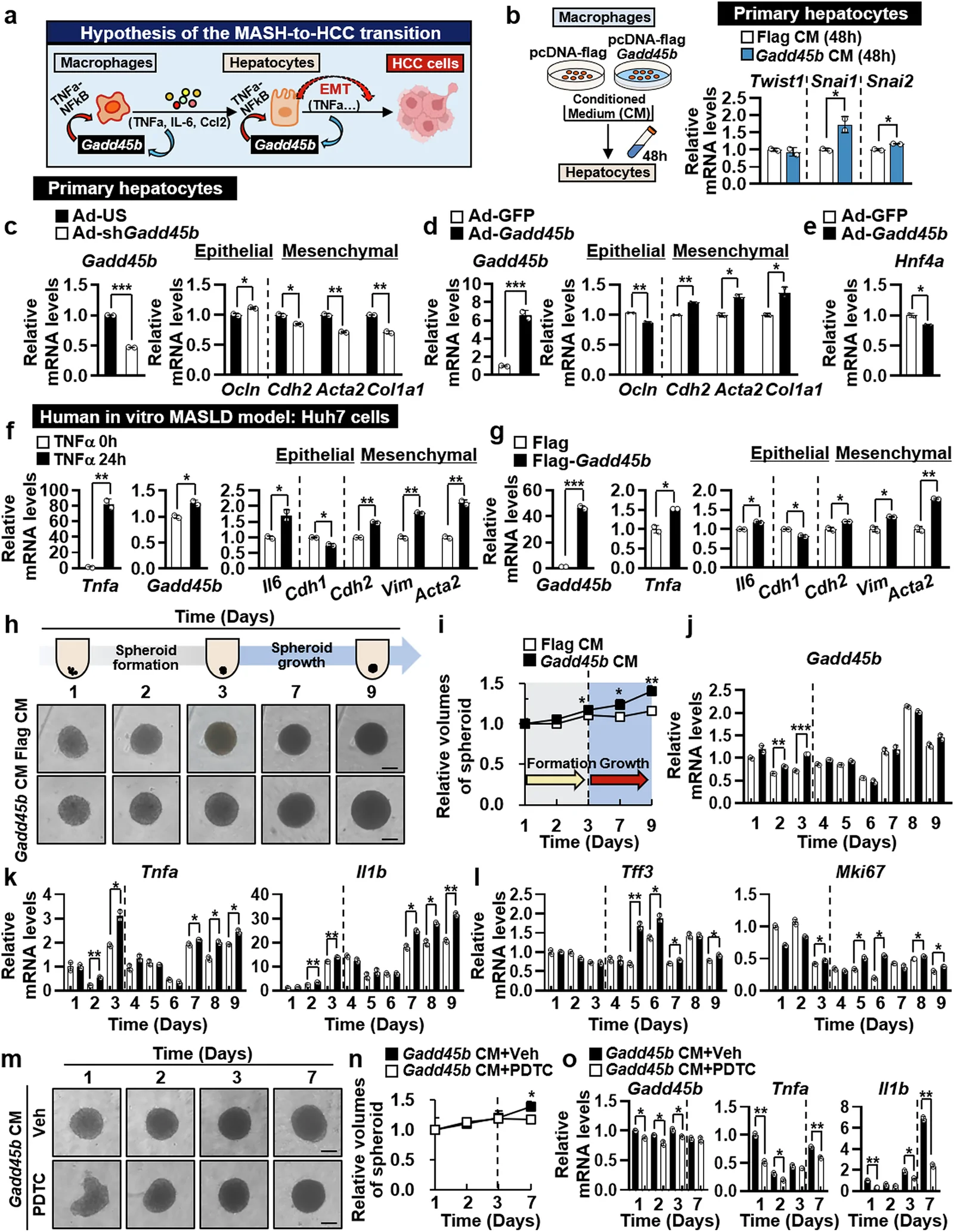
Gadd45b-mediated inflammatory signaling promotes partial EMT-like programs and spheroid formation. **a** Schematic diagram showing the proposed role of macrophage and hepatocyte Gadd45b in MASH-to-HCC transition. **b** RT–qPCR analysis showing the effects of RAW 264.7 cell-derived Flag CM or *Gadd45b* CM on the expression of EMT-associated transcription factors in mouse primary hepatocytes. Mouse primary hepatocytes were cultured with the CM for 48 h. **c**–**e** RT–qPCR analysis showing the effects of *Gadd45b* knockdown (**c**) and overexpression (**d** and **e**) on EMT-related genes and hepatocyte differentiation marker *Hnf4a* in mouse primary hepatocytes. Cells infected with Ad-US, Ad-sh*Gadd45b* (**c**), or Ad-GFP or Ad-*Gadd45b* (**d** and **e**) were treated with 5 ng/ml TGFβ1 for 48 h. **f** RT–qPCR analysis of *Tnfa*, *Gadd45b*, inflammatory and EMT-related genes in Huh7 cells treated with 20 ng/ml TNFα for 24 h. **g** RT–qPCR analysis showing the effects of *Gadd45b* overexpression on inflammatory and EMT-related genes in Huh7 cells. Cells transfected with pcDNA3-flag or pcDNA3-flag-h*Gadd45b* were treated with 20 ng/ml TNFα for 24 h. **h**–**l** Huh7 spheroids were treated with CM derived from RAW 264.7 cells expressing either Flag or Flag-*Gadd45b*, followed by spheroid assays to assess spheroid formation and growth. **h** Representative brightfield images of spheroids on days 1, 2, 3, 7 and 9 (scale bar, 200 μm). **i** Graph showing the changes in spheroid volume in Flag CM (six spheroids) or *Gadd45b* CM (six spheroids). **j**–**l** RT–qPCR analysis of mRNA levels of *Gadd45b* (**j**), inflammatory (**k**) and tumorigenesis markers (**l**) in spheroids at the indicated time points. **m**–**o** Huh7 spheroids treated with *Gadd45b* CM derived from RAW 264.7 cells were additionally treated with 100 μM PDTC, which was replenished together with CM every 2 days. **m** Representative brightfield images of spheroids on days 1, 2, 3 and 7 (scale bar, 200 μm). **n** Graph showing the changes in spheroid volume in *Gadd45b* CM + vehicle (eight spheroids) or *Gadd45b* CM + PDTC (eight spheroids). **o** RT–qPCR analysis of mRNA levels of *Gadd45b* and inflammatory markers in spheroids at the indicated time points. Data in **b**–**g**, **i**–**l**, **n** and **o** are presented as mean ± s.d., analyzed by *t*-test. *P < 0.05, **P < 0.01, ***P < 0.001.

Epithelial-to-mesenchymal transition (EMT) in hepatocytes, driven by transcription factors such as Twist and Snail1/2, contributes to liver fibrosis by generating hepatocyte-derived myofibroblasts^63,64^. It may also facilitate tumor development by increasing hepatocyte plasticity under chronic inflammatory conditions such as MASH^65^. Based on our finding that proinflammatory CM from *Gadd45b*-expressing macrophages induces fibrogenic programs in hepatocytes, we hypothesized that this inflammatory microenvironment also promotes an EMT-associated transcriptional program in hepatocytes, which may contribute to both fibrogenesis and tumorigenic features (Fig. 6a). Indeed, hepatocytes exposed to CM from *Gadd45b*-expressing macrophages exhibited increased expression of the EMT-associated transcription factors *Snail1/2*, suggesting that the inflammatory microenvironment driven by macrophage Gadd45b is associated with EMT-related transcriptional responses in hepatocytes (Fig. 6b). This effect was partially attenuated by *Gadd45b* knockdown in hepatocytes, indicating that hepatocyte Gadd45b-related inflammation contributes to these EMT-associated transcriptional programs (Supplementary Fig. 13a). Consistently, long-term exposure of hepatocytes to immune mediators such as TNFα and transforming growth factor b1 (TGFβ1) can trigger EMT-related genes^31,64^. Long-term treatment of hepatocytes with TNFα not only induced the expression of proinflammatory and fibrogenic genes, including *Gadd45b*, *Ccl2*, *Tgfb1* and *Acta2*, but also increased the expression of the EMT-associated transcription factor *Snail1*, whereas decreasing the expression of *Hnf4a*, a hepatocyte differentiation marker that negatively regulates EMT^66^ (Supplementary Fig. 13b–d). TGFβ1, a potent inducer of EMT that can be induced by TNFα signaling, also upregulated *Gadd45b* and EMT-related genes in hepatocytes, accompanied by reduced *Hnf4a* expression (Supplementary Fig. 13e–g). Knockdown of *Gadd45b* in hepatocytes partly inhibited TGFβ1-induced EMT-associated gene expression, whereas *Gadd45b* overexpression enhanced it, accompanied by reduced *Hnf4a* expression (Fig. 6c–e). Consistent with these observations, Huh7 cells, which are commonly used as a human in vitro MASLD model despite their HCC origin^67,68^, showed increased expression of *Gadd45b*, *Il6* and EMT-related genes following TNFα treatment (Fig. 6f). *Gadd45b* overexpression in Huh7 cells further amplified these responses, supporting its role in promoting EMT-associated transcriptional responses under inflammatory conditions (Fig. 6g). Collectively, these findings in primary hepatocytes suggest that Gadd45b-mediated proinflammatory signaling may contribute to a profibrotic microenvironment associated with a partial EMT-like program.

Given that chronic inflammation promotes a protumorigenic hepatic environment, we further assessed whether *Gadd45b*-mediated inflammatory signaling modulates spheroid-forming capacity in Huh7 cells. CM from *Gadd45b*-expressing macrophages notably enhanced spheroid formation and upregulated inflammatory and tumorigenic genes, whereas CM from *Gadd45b*-knockdown macrophages attenuated spheroid formation and downregulated the expression of these genes (Supplementary Fig. 14a–f). At the experimental endpoint, however, we did not observe a significant change in *Gadd45b* mRNA expression (Supplementary Fig. 14b,e). Therefore, it remained unclear whether *Gadd45b* expression is dynamically regulated during distinct stages of spheroid formation and progression.

As previously reported, three-dimensional spheroids are formed within approximately 3 days and subsequently enter a growth phase during which spheroid volume continues to increase^69,70^. To define the temporal dynamics of *Gadd45b* expression during these processes, we performed additional time-course analyses using more finely divided time points throughout the spheroid formation and growth phases (Fig. 6h–k). We found that CM from *Gadd45b*-expressing macrophages increased *Gadd45b* expression on days 2 and 3 during the spheroid formation phase, coinciding with the initial induction of inflammatory factors (Fig. 6j, k). By contrast, inflammatory factors exhibited a second increase on days 7–9 during the spheroid progression phase, whereas no corresponding increase in *Gadd45b* expression was observed at these later time points (Fig. 6j, k). Notably, the early induction of inflammatory signaling was followed by increased expression of the proliferation markers *Tff3* and *Mki67* after day 3 in cells treated with CM from *Gadd45b*-expressing macrophages, suggesting that macrophage-derived Gadd45b promotes spheroid formation by establishing a proinflammatory environment that supports subsequent proliferative expansion (Fig. 6j–l). Collectively, these findings indicate that Gadd45b-mediated inflammatory signaling primarily contributes to the initiation and establishment of tumor spheroids rather than their later growth phase.

To directly assess whether inhibition of NF-κB activation attenuates macrophage-driven tumorigenic phenotypes, we examined spheroid formation in Huh7 cells exposed to CM from *Gadd45b*-expressing macrophages in the presence or absence of PDTC (Fig. 6m–o and Supplementary Fig. 14g). Importantly, PDTC-mediated inhibition of NF-κB signaling suppressed early *Gadd45b* expression and inflammatory factor induction (days 2–3), resulting in reduced spheroid growth and decreased expression of the proliferation markers *Tff3* and *Mki67* at later stages (Fig. 6m–o and Supplementary Fig. 14g). These findings suggest that early activation of the Gadd45b–TNFα–NF-κB signaling axis plays a critical role in promoting spheroid establishment and subsequent expansion.

### Tumor progression and Gadd45b–TNFα–NF-kB axis in the MASH–HCC model

**Fig. 7:**
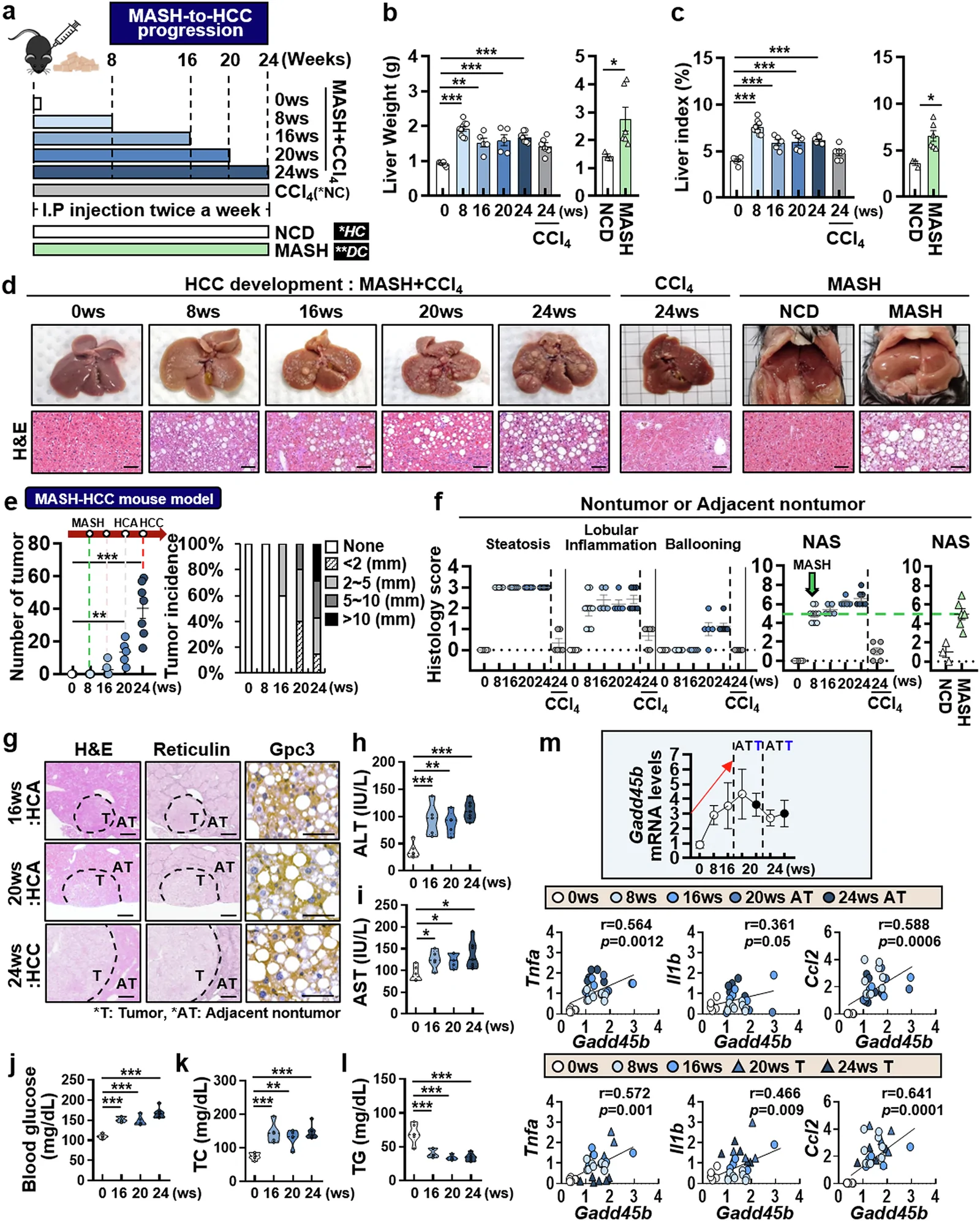
MASH-to-HCC transition is marked by co-upregulation of Gadd45b and inflammatory programs in C57BL6N male mice. **a** Experimental design to determine lesion stages and the Gadd45b–TNFα–NF-kB axis in the progression of MASH to HCC: groups include mice treated with MASH diet + CCl_4_ for 0 (*n* = 6), 8 (*n* = 9), 16 (*n* = 5), 20 (*n* = 5) and 24 weeks (*n* = 7), CCl_4_ (*n* = 6), NCD (*n* = 3) and MASH diet (*n* = 6) alone. **b** Liver weight. **c** Liver index. **d** Representative gross appearance of mouse livers and H&E staining for liver sections of nontumor or adjacent nontumor (400×; scale bar, 50 μm). **e** The number of visible tumors ≥1.0 mm per mouse and the percentages of mice with the indicated maximum tumor sizes. **f** Histology score for steatosis, lobular inflammation, hepatocyte ballooning and NAS. **g** Representative images stained with H&E, reticulin (40×; scale bar, 500 μm) and Gpc3 (400×; scale bar, 50 μm) of liver tumor sections from the MASH diet + CCl_4_ mice at 16, 20 and 24 weeks. **h**–**l** Violin plots showing serum ALT (**h**), AST (**i**), blood glucose levels (**j**), total cholesterol (TC) (**k**) and triglyceride (TG) (**l**) levels at 0, 16, 20 and 24 weeks. The thick dashed line and thin dotted line represent the median and interquartile range. *P < 0.05, **P < 0.01, ***P < 0.001, analyzed by one-way ANOVA followed by Tukey’s test. **m** RT–qPCR analysis of *Gadd45b* mRNA levels and Pearson correlation graphs showing its correlation with the expression levels of inflammatory factors at 0, 8 and 16 weeks, as well as in adjacent nontumor and tumor tissue at 20 and 24 weeks. Pearson’s correlation coefficients (*r*) and *P* values are represented for each analysis. Data in **b**, **c** and **e** are presented as mean ± s.e.m., analyzed by one-way ANOVA followed by Tukey’s test. *P < 0.05, **P < 0.01, ***P < 0.001.

To determine the lesion stage and validate the Gadd45b–TNFα–NF-kB axis in MASH-driven HCC, male C57BL/6N mice were exposed to a MASH diet plus CCl_4_ for 8, 16, 20 and 24 weeks (Fig. 7a). Control groups included CCl_4_ alone and NCD as negative controls, and the MASH diet alone as a disease control (Fig. 7a). MASH diet+CCl_4_ mice showed increased liver weight and liver-to-body weight ratio from 8 weeks, similar to the MASH diet alone group, and maintained these elevations throughout tumorigenesis (Fig. 7b, c). Multiple liver nodules appeared from 16 weeks, and tumors were observed in all mice at 20 and 24 weeks (Fig. 7d, e). The NAS at 8 weeks was comparable to that of the MASH diet group and progressively increased during tumorigenesis, whereas the CCl_4_-alone group showed no significant changes, suggesting that MASH is a key driver of tumor development in this model (Fig. 7d, f).

Based on reticulin loss and cytoplasmic Gpc3 expression, tumors at 16 and 20 weeks were classified as HCA, whereas those at 24 weeks were identified as HCC by an expert pathologist (Fig. 7g). *Tff3*, a regulator of HCC proliferation, showed a clear and progressive induction from 16 to 24 weeks (Supplementary Fig. 15a). Serum ALT, AST, glucose and total cholesterol levels gradually increased during tumorigenesis, whereas circulating TG levels decreased (Fig. 7h–l). The Lpl–Fabp4–Cpt1c axis, a well-established marker of MASH-derived HCC, was progressively upregulated across lesion stages, supporting that tumor in this model arise from MASH (Supplementary Fig. 15b). In this model, Gadd45b, identified as a novel driver of MASH-to-HCC progression, was progressively upregulated during tumorigenesis and showed a positive correlation with the proinflammatory genes (Fig. 7m and Supplementary Fig. 15c). Lesion stages were defined as MASH (8 weeks), advanced MASH (16 weeks), HCA (20 weeks) and HCC (24 weeks), based on the NAS score and tumor development (Supplementary Fig. 15d).

To examine the sex-specific relevance of the novel MASH–HCC marker, female C57BL/6N mice were analyzed under the same conditions (Supplementary Fig. 16). Tumor development and molecular changes in female mice closely mirrored those in male mice, suggesting that the MASH–HCC model and the Gadd45b–TNFα–NF-kB axis are broadly applicable across sexes and may serve as a robust preclinical platform (Supplementary Fig. 16).

### Pharmacological validation of the MASH–HCC model and its novel progression markers

**Fig. 8:**
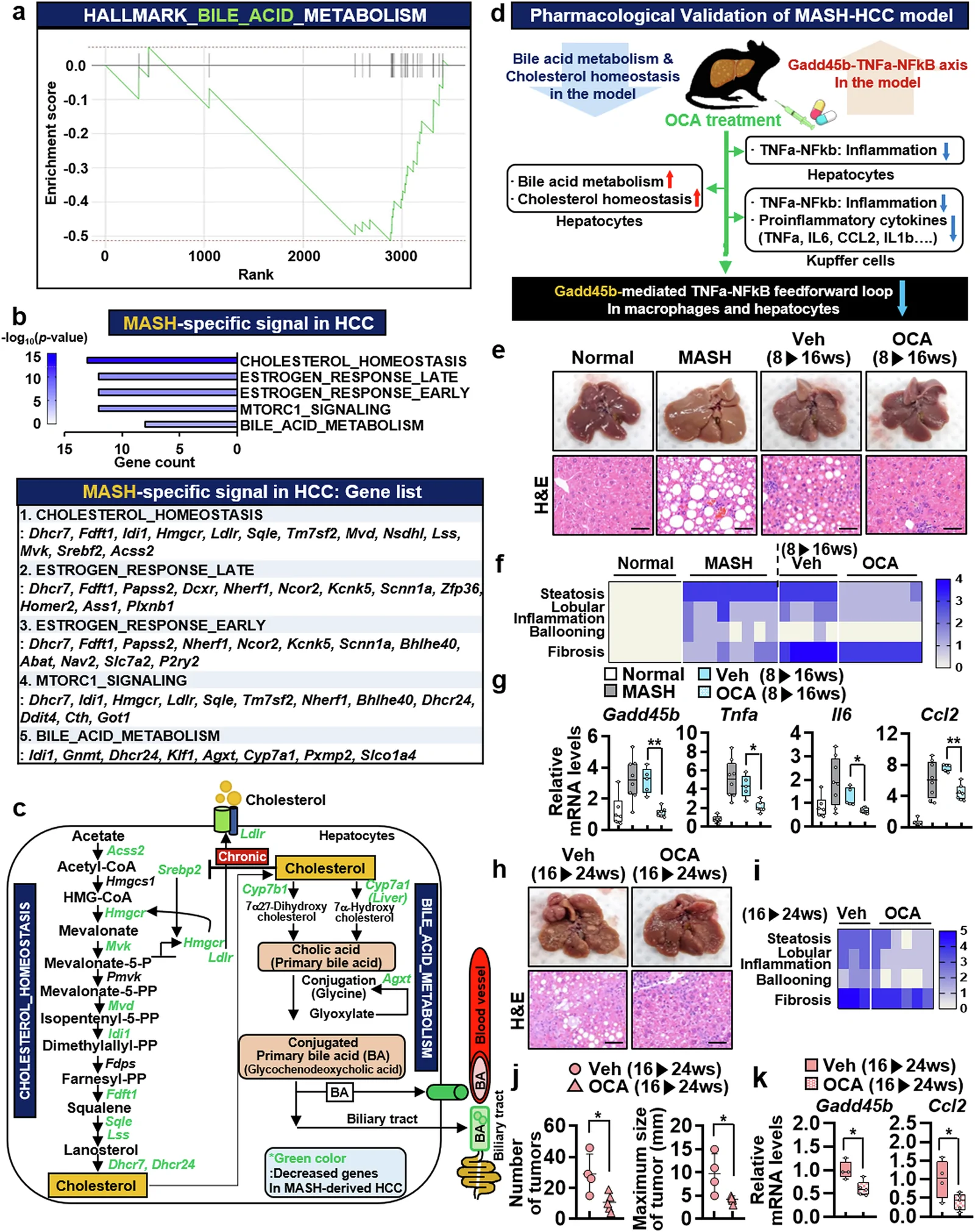
Therapeutic intervention reverses the Gadd45b–TNFα–NF-kB axis during MASH-to-HCC progression. **a** Enrichment plots for Hallmark gene set by GSEA. GSEA identified the ‘HALLMARK BILE ACID METABOLISM’ gene set as downregulated in the HCC mouse model (MASH+CCl_4_^high^_T versus NCD). **b** Hallmark pathway enrichment of downregulated genes associated with MASH in HCC (MASH+CCl_4_^high^_T versus CCl_4_^high^) visualized on a bar chart, showing number of shared genes (count), −log_10_ (FDR *q* value), and gene lists. **c** A schematic summary illustrates the cholesterol and bile acid metabolism pathways in hepatocytes. Key genes involved in cholesterol biosynthesis, such as *Acss2*, *Hmgcr*, *Mvk*, *Mvd*, *IdI1*, *Fdft1*, *Sqle*, *Lss*, *Tm7sf2*, *Nsdhl*, *Sc5d*, *Dhcr7* and *Dhcr24*, as well as those involved in bile acid synthesis, including *Cyp7b1*, *Cyp7a1*, *Agxt* and *Slco1a4*, are shown. Green color indicates decreased expression in the MASH-derived HCC mouse model. **d** Schematic summary illustrating the therapeutic effects of OCA in the refined MASH–HCC mouse model. The diagram highlights the potential modulation of the Gadd45b–TNFα–NF-kB axis upon OCA treatment and explores the hypothesis that Gadd45b may act as a therapeutic target by regulating a TNFα–NF-kB-driven inflammatory feed-forward loop in macrophages and hepatocytes. **e** Representative gross appearance of mouse livers and H&E staining (400×; scale bar, 50 μm) in the livers from mice treated with NCD, MASH, vehicle or OCA from 8 to 16 weeks. **f** Heatmap analysis showing hepatic scores for steatosis, lobular inflammation, hepatocyte ballooning and fibrosis. **g** RT–qPCR analysis of *Gadd45b*, and inflammatory markers in the liver tissues of mice. The box plot shows data distribution. *P < 0.05, **P < 0.01, analyzed by one-way ANOVA followed by Tukey’s test. **h** Representative gross appearance of mouse livers and H&E staining (400×; scale bar, 50 μm) in the livers from mice treated with vehicle or OCA from 16 to 24 weeks. **i** Heatmap analysis showing hepatic scores for steatosis, lobular inflammation, hepatocyte ballooning and fibrosis. **j** The number of visible tumors ≥1.0 mm per mouse and the maximum size of tumors per mouse. **k** RT–qPCR analysis of *Gadd45b* and *Ccl2* mRNA levels in tumor tissues from mice treated with vehicle or OCA from 16 to 24 weeks. The box plot shows data distribution. *P < 0.05, analyzed by *t*-test. Data in **j** are presented as mean ± s.e.m., analyzed by *t*-test. *P < 0.05. FDR, false discovery rate.

To determine whether the molecular markers identified in our model reflect disease severity and are reversible upon therapeutic intervention, we examined the most prominently downregulated pathways in this model (Fig. 8a). Gene set analysis revealed that pronounced suppression of bile acid- and cholesterol-related signaling in this model (Fig. 8a–c). BILE_ACID_METABOLISM emerged as the most significantly downregulated KEGG pathway, whereas CHOLESTEROL_HOMEOSTASIS showed the strongest downregulation among pathways associated with MASH (Fig. 8b, c). Genes contributing to these pathways are indicated in Fig. 8c. These metabolic disturbances suggest that bile acid dysregulation in our model is caused by impaired cholesterol metabolism, which constitutes a MASH-specific metabolic feature (Fig. 8c). These findings led us to select OCA, a selective FXR agonist, for pharmacological validation. OCA targets dysregulated hepatic bile acid and cholesterol metabolism and inhibits TNFα-induced NF-kB activation in hepatocytes and KCs, thereby attenuating liver inflammation, fibrosis and HCC development^71,72^ (Fig. 8d). Given that OCA targets both the downregulated bile acid/cholesterol metabolism and the upregulated TNFα–NF-kB signaling in this model, it was selected for pharmacological validation of the MASH–HCC model and its molecular markers (Fig. 8d). OCA was administrated at the MASH (8 weeks) and advanced MASH (16 weeks) stages to evaluate its ability to prevent disease progression (Fig. 8e–k and Supplementary Fig. 17a). At both stages, OCA treatment markedly improved histological features of steatosis, lobular inflammation, ballooning and fibrosis (Fig. 8e,f,h,i). Notably, OCA treatment at the advanced MASH stage also suppressed tumor formation (Fig. 8j). At the molecular levels, OCA reduced the expression of *Gadd45b* and inflammatory genes, highlighting the Gadd45b–TNFα–NF-kB axis as a key marker of MASH-to-HCC progression (Fig. 8g, k).

Co-immunostaining analyses further revealed that OCA treatment reduced Gadd45b expression in HNF4a-positive hepatocytes and F4/80-positive macrophages at both disease stages (Supplementary Fig. 17b,c). By contrast, Gadd45b/Desmin-double-positive cells were rarely detected (Supplementary Fig. 17b,c). These findings suggest that hepatocytes and macrophages are the primary cellular targets associated with the OCA-mediated suppression of Gadd45b expression in the liver.

## Discussion

MASH has rapidly emerged as a major cause of HCC. However, the mechanisms driving the progression from MASH to HCC and the potential therapeutic targets remain limited. In this study, despite the use of a CCl_4_-based protocol, careful modulation of metabolic damage and fibrosis through MASH-inducing diet and mouse strain selection enabled the establishment of a MASH-derived HCC model in which MASH-associated metabolic stress serves as the primary driver of tumorigenesis. This model recapitulates the full histopathological spectrum, progressing from MASH (8 weeks) to advanced MASH (16 weeks), HCA (20 weeks) and HCC (24 weeks)—all within 24 weeks. This progression is much faster than that of other C57BL/6N-based models, which require up to 40 weeks to induce HCC^18^. The resulting HCC was histologically distinct from benign lesions and molecularly validated by upregulation of the Lpl–Fabp4–Cpt1c axis^48^, a known marker of MASH-derived HCC. Unlike many existing models in which tumorigenesis is driven predominantly by genetic manipulation or severe toxic injury, our approach preserves the contribution of chronic metabolic stress and inflammation as central drivers of disease progression. This feature provides a unique opportunity to investigate stage-specific molecular events underlying hepatocarcinogenesis and to evaluate preventive or therapeutic interventions.

A central finding of this study is the identification of Gadd45b as a previously unrecognized potentially actionable regulator linking chronic inflammation and hepatocarcinogenesis. Rather than functioning merely as a marker of disease progression, Gadd45b actively orchestrated inflammatory crosstalk between macrophages and hepatocytes, thereby establishing a permissive microenvironment for tumor initiation.

Another notable strength is the concordance observed between human and mouse datasets. The stage-dependent increase in *GADD45B*/*Gadd45b* expression, inflammatory activation and macrophage remodeling was consistently detected across human transcriptomic datasets, human single-cell analyses and our mouse model. This cross-species consistency strengthens the translational relevance of our findings and supports the likelihood that the identified Gadd45b-dependent inflammatory circuitry reflects a conserved mechanism of MASH-associated hepatocarcinogenesis. Mechanistically, Gadd45b amplified liver inflammation through a TNFα–NF-κB feed-forward loop between macrophages and hepatocytes, thereby promoting tumorigenic transcriptional programs and HCC development. Importantly, the dynamic regulation of Gadd45b during disease progression and following pharmacological intervention further supports its role in inflammation-driven hepatocarcinogenesis.

However, this study has several limitations. The model relies on a specific combination of dietary and chemical induction. Although we modified the CCl_4_-based protocol^16–18^ to favor the metabolic environment over toxic injury, CCl_4_ may still influence disease progression. Moreover, while C57BL/6N mice were selected for their susceptibility to metabolic injury and attenuated responses to chemical-induced excessive fibrosis^16–18,22,23^, validation in additional strains and models will be important to capture the heterogeneity of human MASH-to-HCC progression. Encouragingly, disease progression and tumorigenesis were consistent across sexes, supporting the broader applicability of this model.

Another challenge of this study is that cell-type-specific genetic models were not used. Although our findings, together with co-immunostaining analyses and adenoviral knockdown studies, support distinct roles for macrophage and hepatocyte Gadd45b in the initiation and amplification of inflammatory signaling, macrophage-specific and hepatocyte-specific *Gadd45b* deletion models will be required to definitively establish their compartment-specific functions in vivo.

Collectively, our findings position Gadd45b as a pivotal driver of the MASH-to-HCC transition. By promoting macrophage–hepatocyte crosstalk, Gadd45b contributes to the establishment of a protumorigenic liver microenvironment and represents a potential therapeutic target in MASH-associated HCC. Furthermore, our refined model provides a robust and time-efficient platform for mechanistic studies and preclinical therapeutic evaluation in MASH-driven hepatocarcinogenesis.

## Supplementary information

Supplementary Information

Supplementary Data 1

## Data Availability

Data that supported the findings of this study are available from the corresponding author upon request. Reagents, resources and protocols are included in the ‘Materials and methods’ section or the Supplementary Information. RNA-seq data have been deposited into the National Center for Biotechnology Information Gene Expression Omnibus under accession number GSE286552 (www.ncbi.nlm.nih.gov/geo/query/acc.cgi?acc=GSE286552).
