# Supplementary material for: A refined MASH–HCC model identifies macrophage Gadd45b as a key orchestrator of inflammation-driven neoplastic progression
Source: Exp Mol Med. 2026 Sep 16;58(9):3196–213. doi: 10.1038/s12276-026-01838-5 (PMC13633257; doi:10.1038/s12276-026-01838-5)
Supplement: Supplementary file 1 [file 12276_2026_1838_MOESM1_ESM.pdf]

Supplementary Information

A refined MASH-HCC model identifies macrophage Gadd45b as a key orchestrator of inflammation-driven neoplastic progression

Hyunmi Kim[1†], **Gyeonghun Kim**[2,3†], Hyo Jin Yeon[4], Da-Yeon Yang[1,5], Sang Gyu Lee[2,3,6], Tae Hyeon An[1], Seung Yeon Oh[2,3], Seona Yoon[3], Jiwoo Kim[4], Juyong Choi[1,5], Hyun-Ju Park[1,5], Eun-Woo Lee[1,5], Baek-Soo Han[5,7], Chul-Ho Lee[5,8], Il Yong Kim[2,3], Won Kon Kim[1,5], Kwang-Hee Bae[1,5], Jun Won Park[9], Seung Hyun Oh[10], Young Nyun Park[11], Murim Choi[4], **Je Kyung Seong**[2,3,6,12,13]* and **Kyoung-Jin Oh**[1,5]*

Affiliations:
[1]Metabolic Regulation Research Center, Korea Research Institute of Bioscience and Biotechnology (KRIBB), Daejeon, Republic of Korea
[2]Laboratory of Developmental Biology and Genomics, College of Veterinary Medicine, Seoul National University, Seoul, Republic of Korea
[3]Korea Model animal Priority Center, Seoul National University, Seoul, Republic of Korea
[4]Department of Biomedical Sciences, Seoul National University College of Medicine, Seoul 03080, Republic of Korea
[5]Department of Functional Genomics, KRIBB School of Bioscience, Korea University of Science and Technology (UST), Daejeon, Republic of Korea
[6]Interdisciplinary Program in Bioinformatics, Seoul National University, Seoul, Republic of Korea
[7]Biodefense Research Center, Korea Research Institute of Bioscience and Biotechnology (KRIBB), Daejeon, Republic of Korea
[8]Laboratory Animal Resource Center, Korea Research Institute of Bioscience and Biotechnology (KRIBB), Daejeon, Republic of Korea
[9]Laboratory Animal Medicine, College of Veterinary Medicine, Seoul National University, Seoul, Republic of Korea
[10]Laboratory of Veterinary Histology, College of Veterinary Medicine, Seoul National University, Seoul, Republic of Korea
[11]Department of Pathology, Severance Hospital, Yonsei University College of Medicine, Seoul, Korea.
[12]Interdisciplinary Program of Cancer Biology, Seoul National University Cancer Research Institute, Seoul, Republic of Korea.
[13]BIO MAX/N-Bio Institute, Seoul National University, Seoul, Republic of Korea
[†]These authors contributed equally: Hyunmi Kim, Gyeonghun Kim

Running title: Role of macrophage Gadd45β in MASH-HCC progression

***Corresponding authors:**

Kyoung-Jin Oh, Metabolic Regulation Research Center, KRIBB, Daejeon 34141, Republic of Korea; Tel: +82-42-879-8265; E-mail: kjoh80@kribb.re.kr

Je Kyung Seong, College of Veterinary Medicine, Seoul National University, Seoul 08826, Republic of Korea; Tel: +82-2-880-1259; E-mail: snumouse@snu.ac.kr

Supplementary Figures and Figure Legends

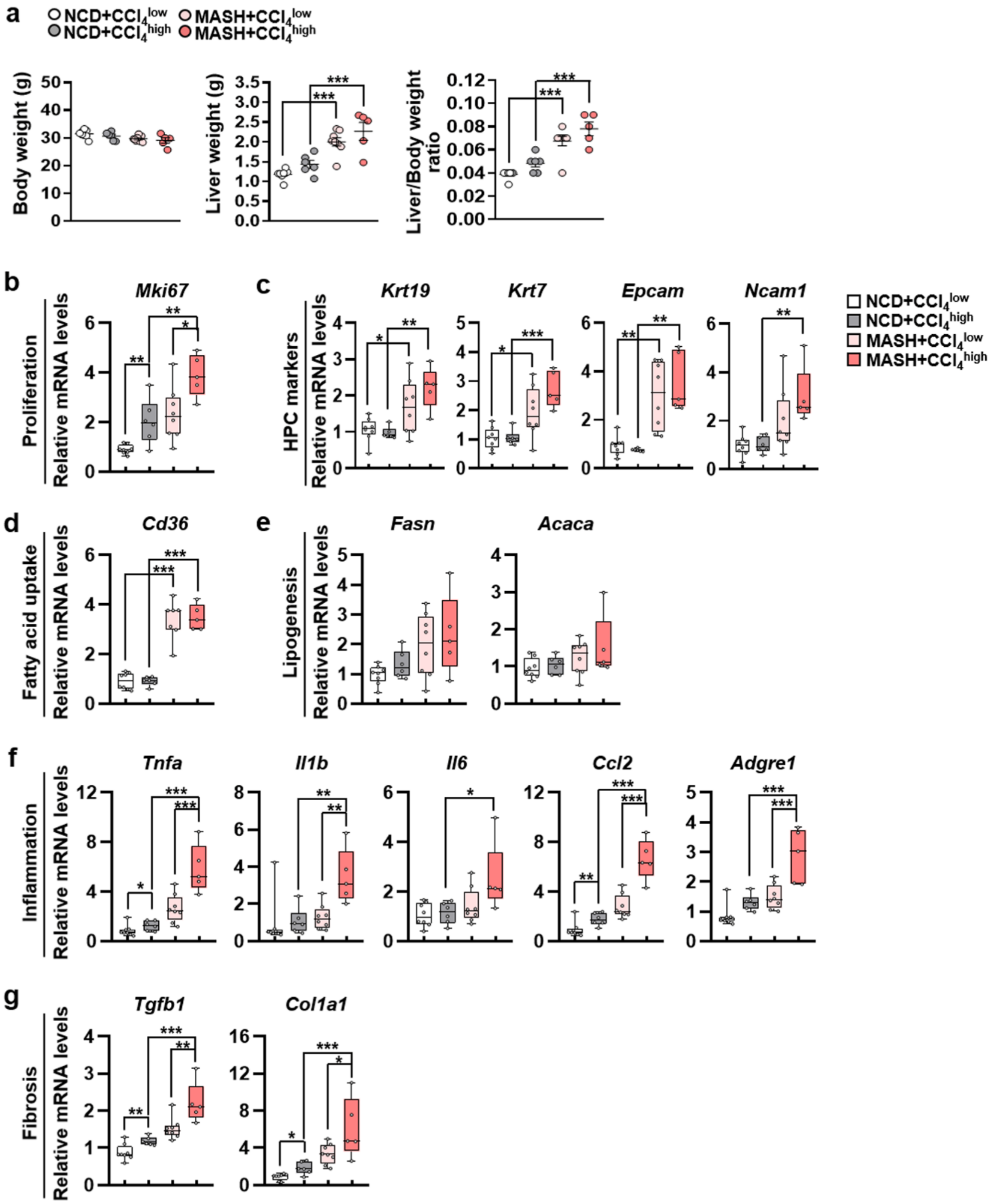

Supplementary Fig. 1. Effects of a MASH-provoking diet on liver tumorigenesis in C57BL/6N mice under controlled fibrotic stress

a–g a MASH-associated HCC mouse model: Groups include CCl_4^{low} (n = 8), CCl_4^{high} (n = 6), MASH diet + CCl_4^{low} (n = 8) and MASH diet + CCl_4^{high} (n = 5). **a** Body weight, liver weight, and liver/body weight ratio at 24 weeks. **b–g** RT-qPCR analysis of a proliferation marker (**b**), HPC markers (**c**), a fatty acid uptake marker (**d**), lipogenesis markers (**e**), inflammatory markers (**f**), and fibrotic markers (**g**) in mouse liver tumors. The box plots show the distribution of data based on its five-number summary (minimum, lower quartile, median, upper quartile,

and maximum). $^{*}P<0.05$, $^{**}P<0.01$, $^{***}P<0.001$, analyzed by one-way ANOVA followed by Tukey's test. Data in (**a**) is presented as mean ± SEM, analyzed by one-way ANOVA followed by Tukey's test, $^{***}P<0.001$.

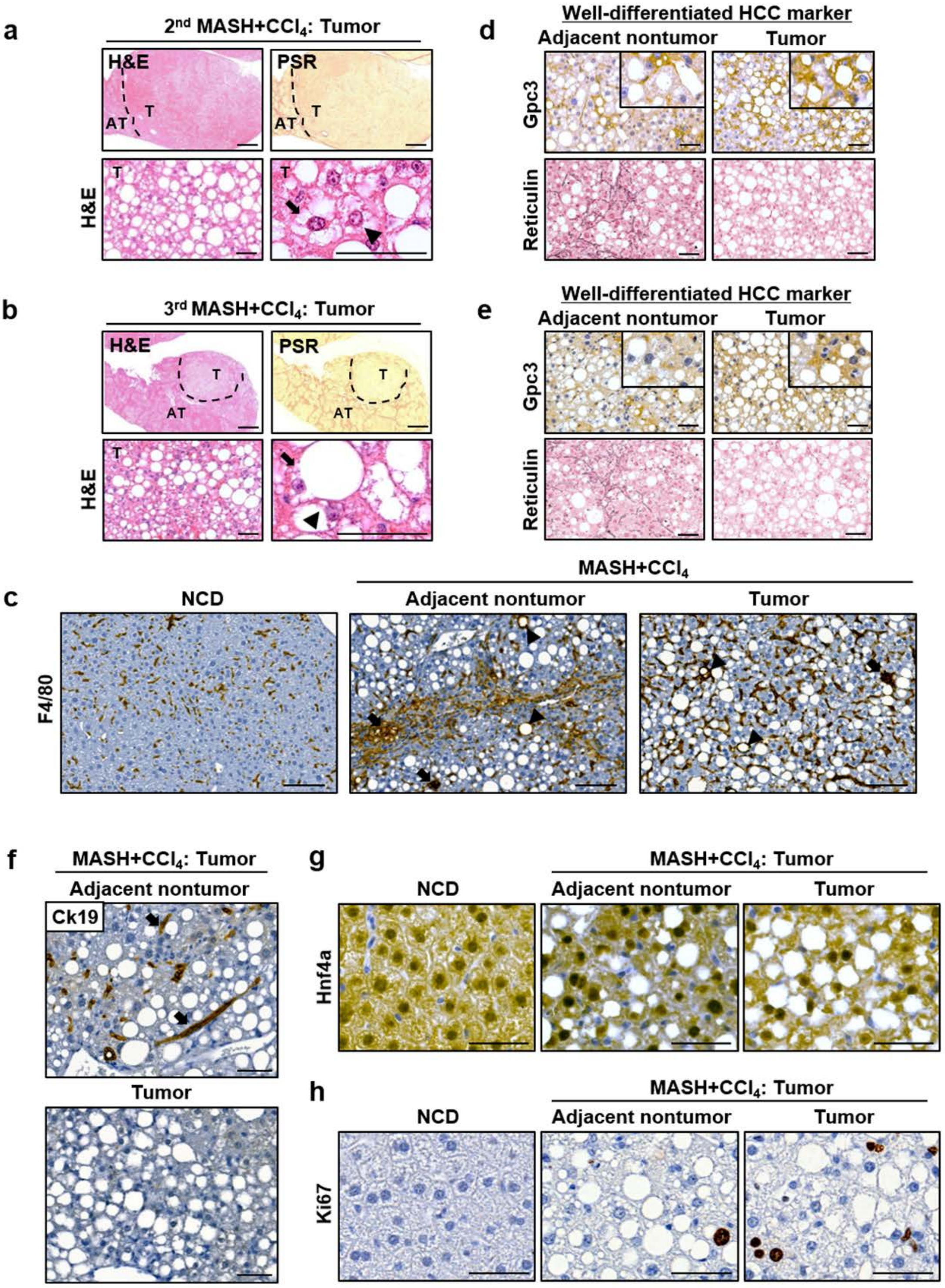

Supplementary Fig. 2. Histopathological characteristics of liver tumors induced by a MASH-provoking diet in C57BL/6N mice under controlled fibrotic stress

a, b Representative H&E and Picrosirius Red (PSR) staining of liver tumor sections from MASH diet + $CCl4^{high}$ mice (20×, scale bar = 1 mm), shown from the second (**a**) and third (**b**) of three independent experiments. Higher magnification images (400×, scale bar = 50 μm) show sections stained with H&E. In the H&E sections, hepatocyte ballooning (arrow) and Mallory-Denk bodies (arrowhead) are visible. **c** Immunohistochemistry (IHC) staining for

F4/80 in liver tissues from NCD, adjacent nontumor and tumor sections of MASH diet + CCl_4^{high} mice (200×, scale bar = 100 μm), showing inflammatory foci (arrows) and hepatic crown-like structures (arrowheads). **d, e** Representative Gpc3 and Reticulin (for fiber visualization) staining of liver tumor sections from MASH diet + CCl_4^{high} mice (400×, scale bar = 50 μm), shown from the second (**d**) and third (**e**) of three independent experiments. The inset in the Gpc3 panel clearly shows the hepatocyte nucleus and cytoplasm. **f** IHC staining for Ck19, a cholangiocyte marker, showing ductular reaction (arrows) (400×, scale bar = 50 μm). **g, h** IHC staining for Hnf4a (**g**) and Ki67 (**h**) in liver tissues from NCD, adjacent nontumor and tumor sections of MASH diet + CCl_4^{high} mice (400×, scale bar = 50 μm).

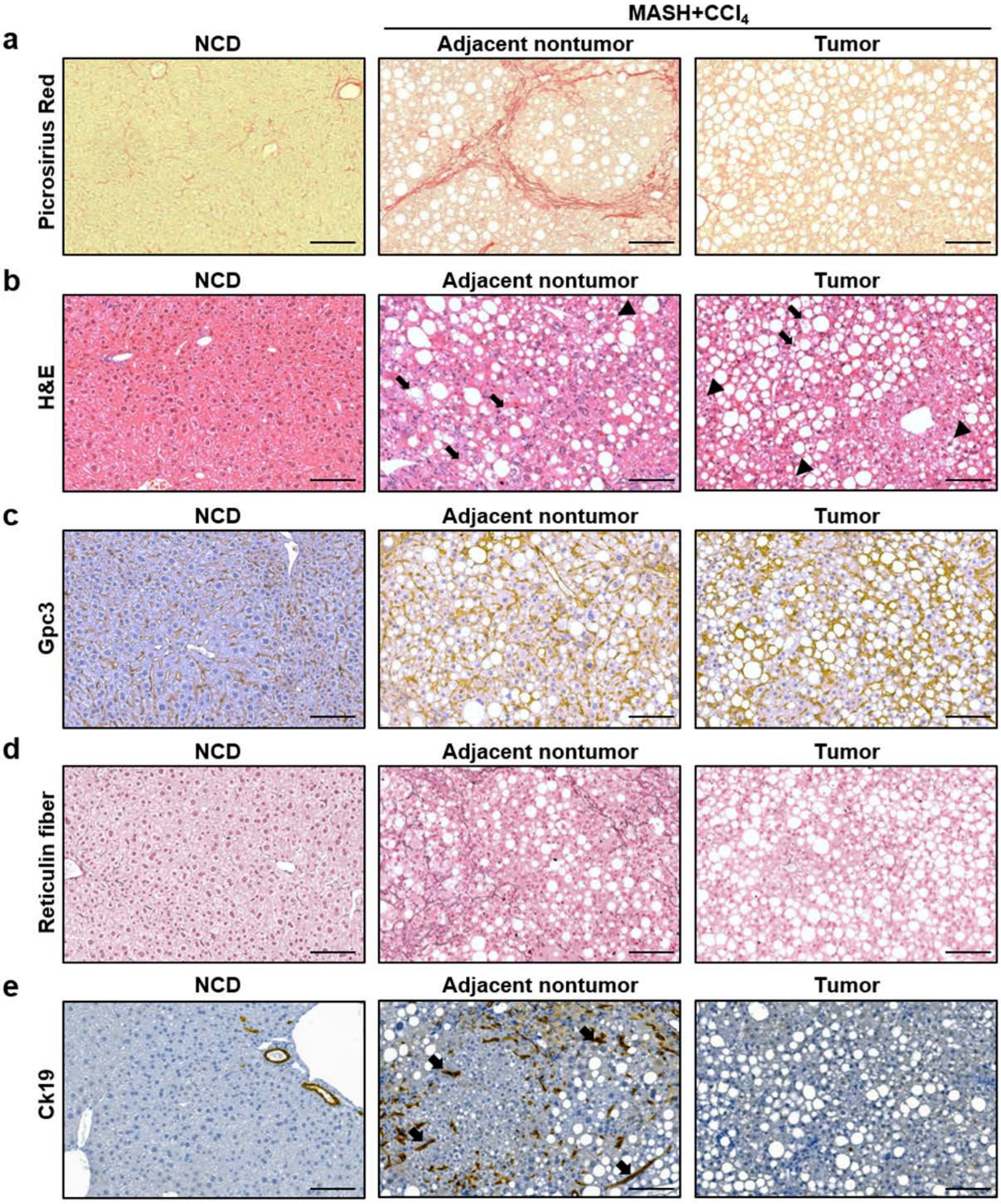

Supplementary Fig. 3. Histological comparison of livers from normal chow-fed mice and tumor-adjacent and tumor tissues in diet-driven MASH-associated hepatocarcinogenesis. **a–e** Representative histology of liver tissue from NCD mice, adjacent-to-tumor and tumor tissues from the MASH diet+CCl4high mice (200×, scale bar = 100 µm). **a** Picrosirius Red staining of liver tissue. **b** H&E staining of liver tissue. The image highlights key morphological features such as steatosis, hepatocyte ballooning (arrows), and Mallory-Denk bodies (arrowheads). **c** IHC staining for Gpc3. **d** Reticulin staining of liver tissue. **e** IHC staining for Ck19, a cholangiocyte marker, showing ductular reaction (arrows).

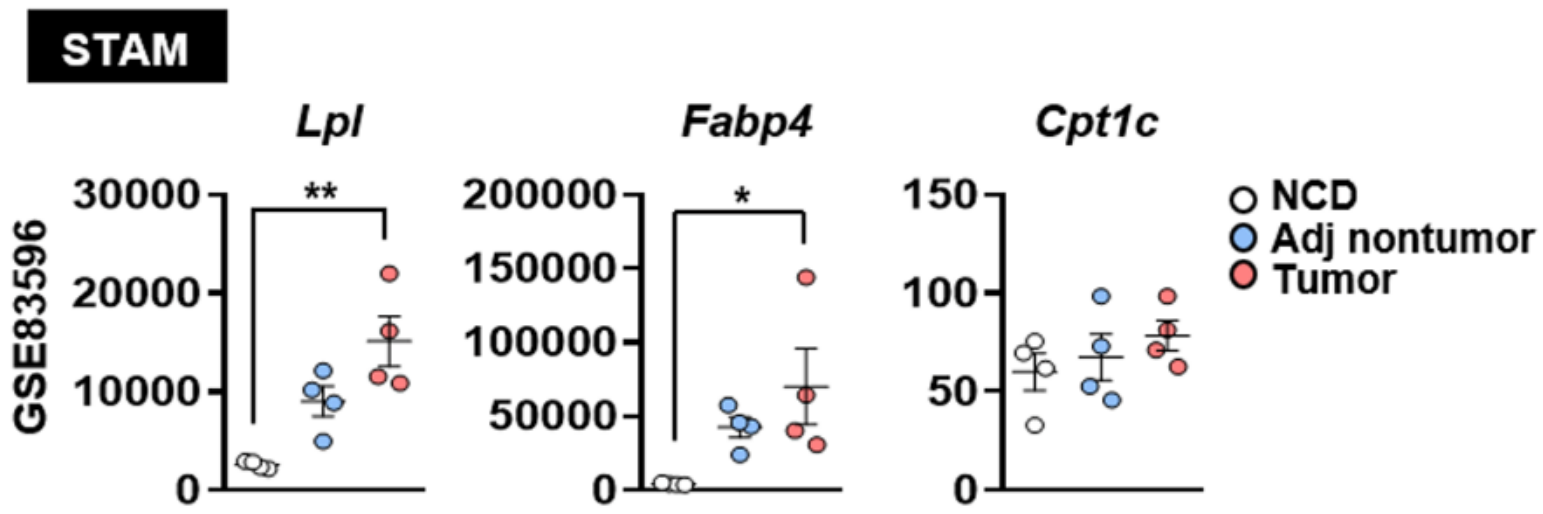

Supplementary Fig. 4. Expression pattern of the Lpl-Fabp4-Cpt1c axis in the STAM mouse model.

The expression pattern of the Lpl-Fabp4-Cpt1c axis was analyzed using the GEO dataset (GSE83596).

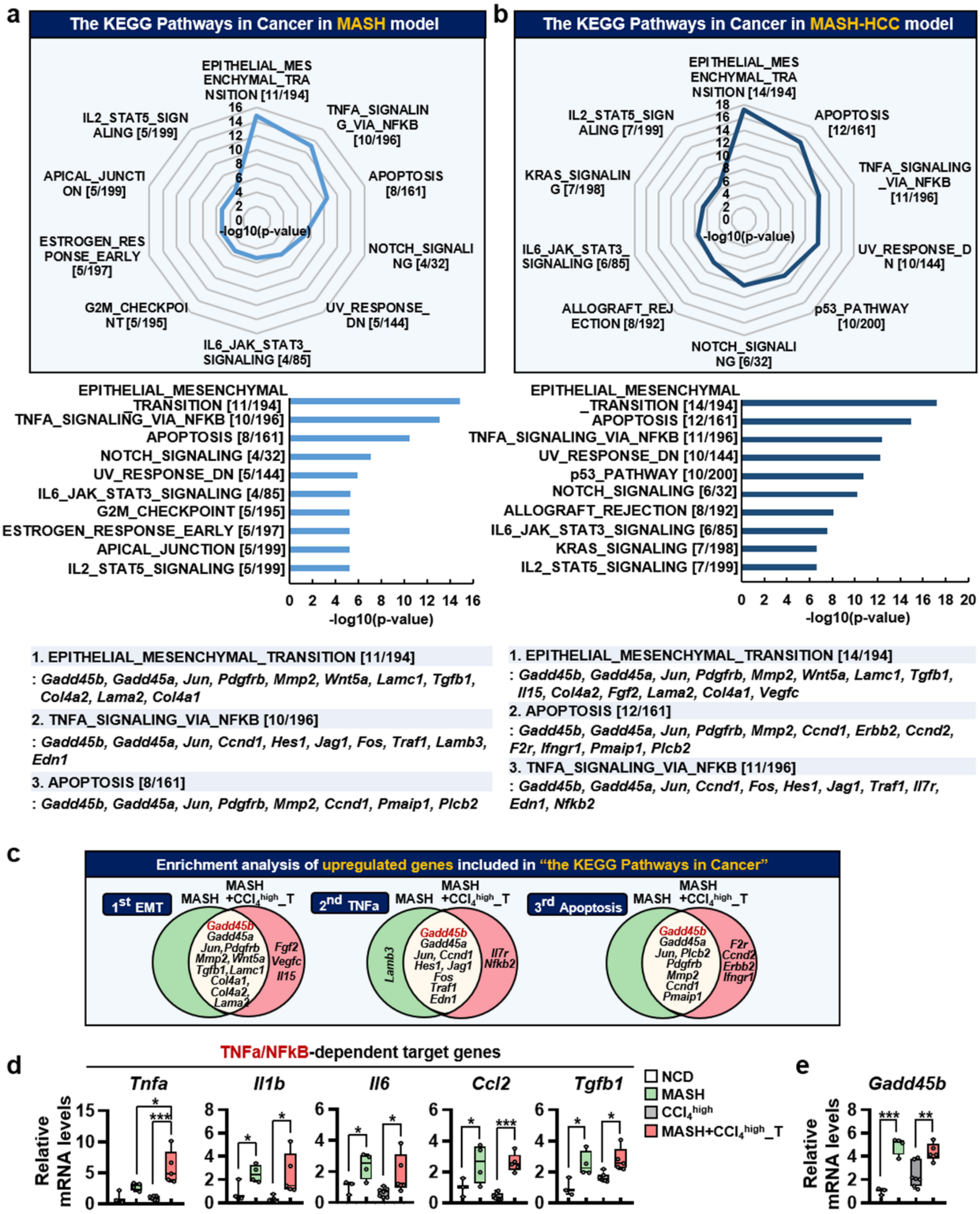

Supplementary Fig. 5. Hallmark gene set enrichment analysis of upregulated genes included in the KEGG Pathways in Cancer in MASH and MASH-HCC models.

a, b Radial line graphs and bar charts showing Hallmark gene set enrichment of upregulated genes involved in KEGG Pathways in Cancer in the MASH model (**a**) and tumors from the MASH–HCC model (**b**), along with associated gene lists. Bracketed values represent overlap/input gene numbers. **c** Venn diagram showing three commonly upregulated pathways in the MASH and MASH-HCC groups, along with the corresponding lists of intersecting genes. **d, e** RT-qPCR analysis of TNFa-NFkB downstream target genes (**d**) and *Gadd45b* (**e**) in mouse

livers from the NCD (n = 3), MASH (n = 4), CCl_4^{high} (n = 7), and HCC (MASH diet + CCl_4^{high}, n = 4) groups. The box plot shows data distribution. $^{*}P<0.05$, $^{**}P<0.01$, $^{***}P<0.001$, analyzed by one-way ANOVA followed by Tukey's test.

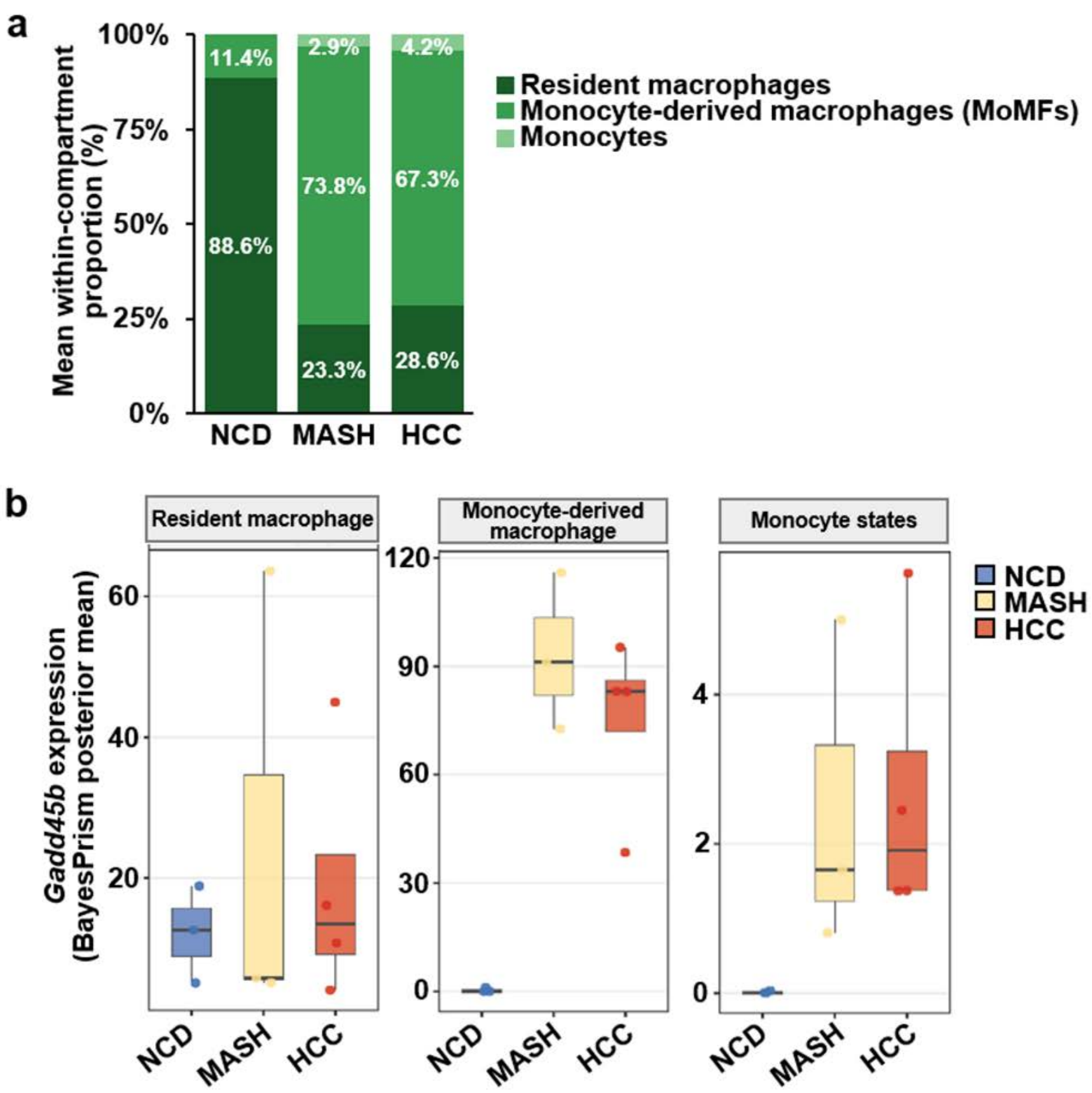

Supplementary Fig. 6. Gadd45b expression tracks stage-dependent remodeling of liver macrophage subsets during MASH-to-HCC progression in mice.

a, b BayesPrism deconvolution of mouse liver bulk RNA-seq data using a mouse sc/snRNA-seq reference dataset (GSE192742). **a** Bar graph showing disease stage-dependent changes in macrophage subtype proportions. **b** The box plot showing *Gadd45b* expression across disease stages and macrophage subtypes.

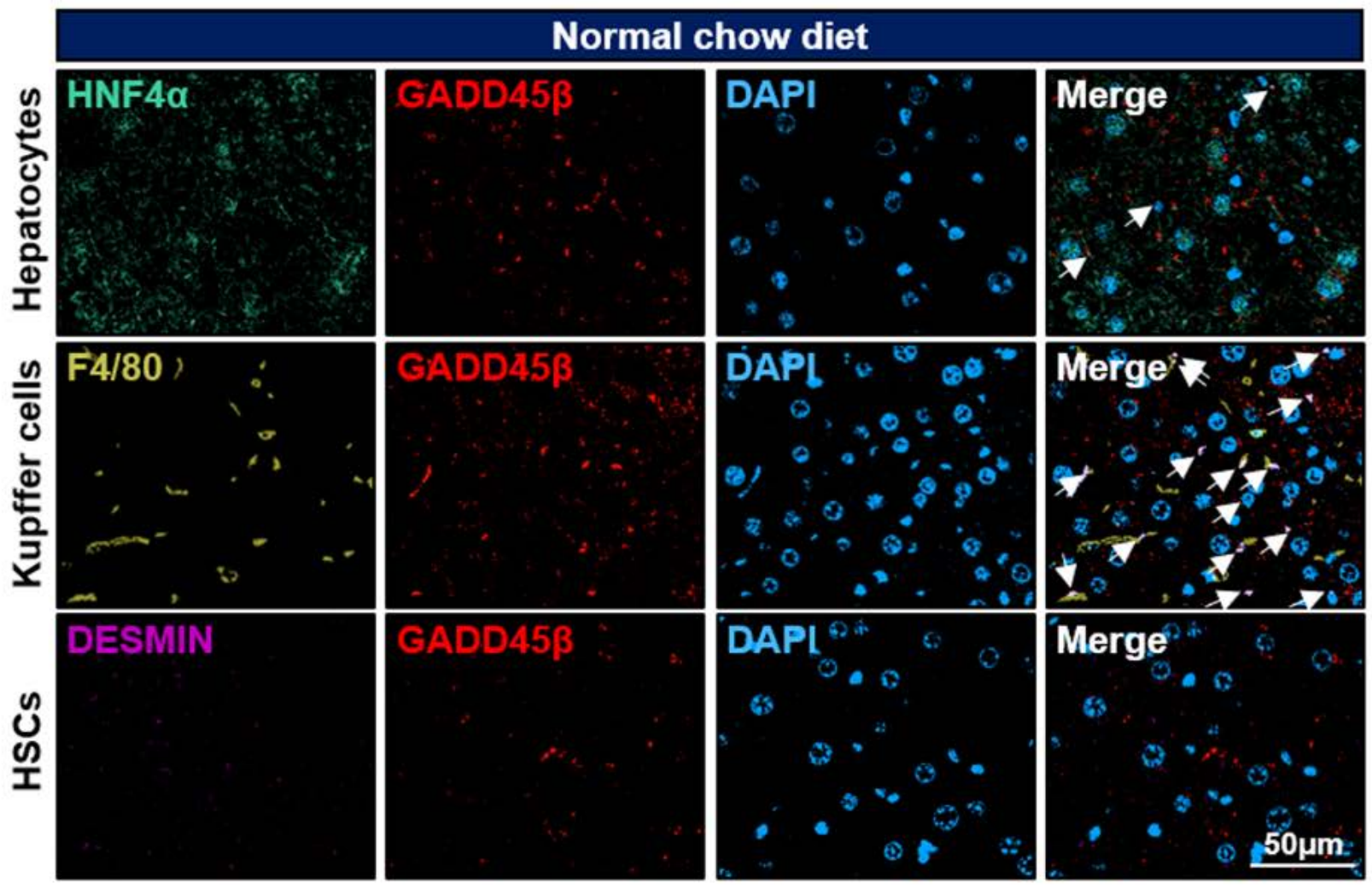

Supplementary Fig. 7. Expression of Gadd45b in the liver under the normal chow conditions.

Immunofluorescence staining of liver sections from the NCD group. Representative images showing co-localization of HNF4α (cyan), F4/80 (yellow), DESMIN (magenta), and Gadd45β (red). Nuclei were counterstained with DAPI (blue) (200×, scale bar = 50 μm).

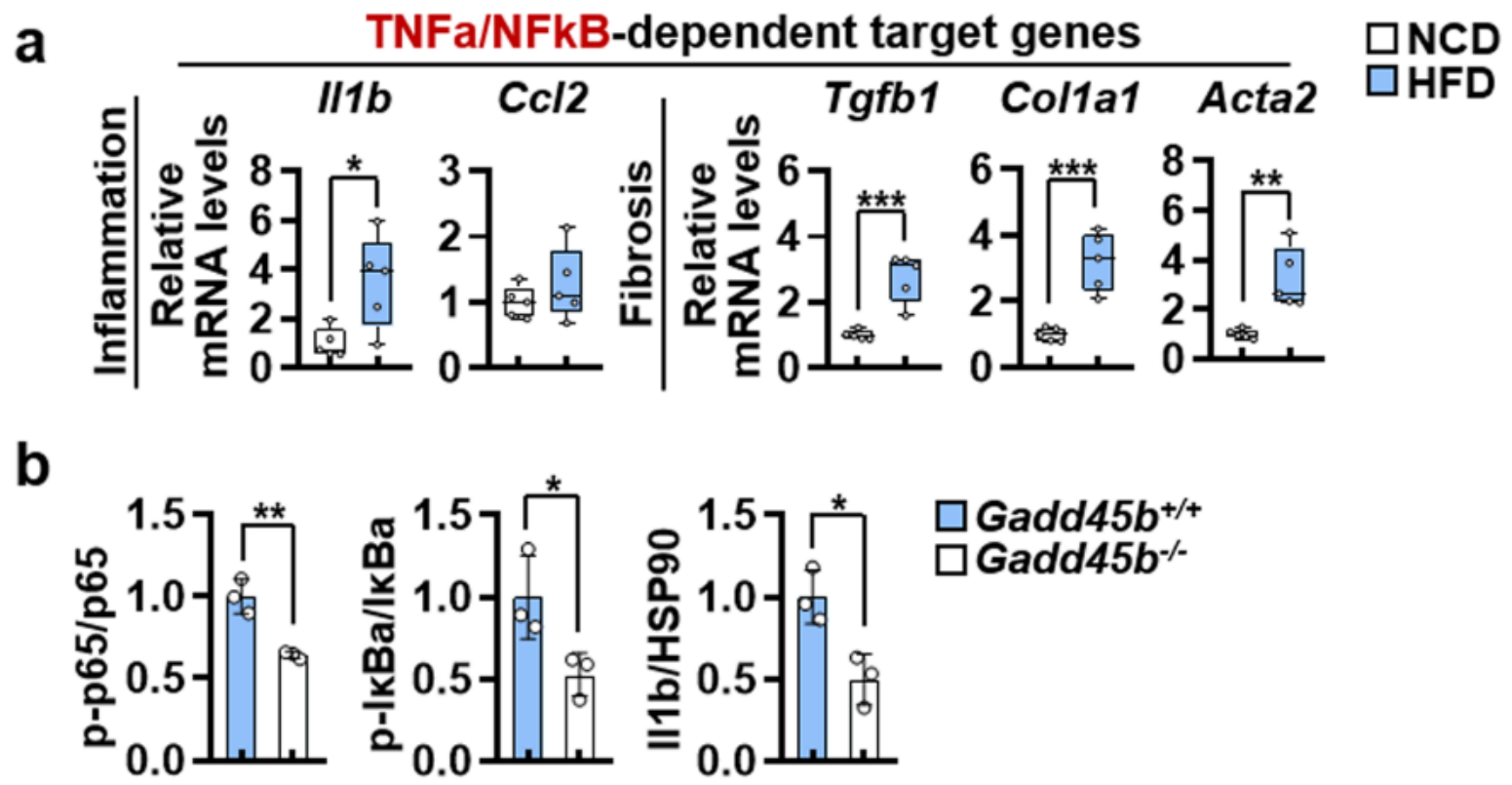

Supplementary Fig. 8. Effects of *Gadd45b* deficiency on TNFa-NFkB signaling under HFD conditions.

a RT-qPCR analysis of TNFa-NF-kB target genes in the livers of mice fed either an NCD or HFD for 12 weeks (n = 5 per group). The box plot shows data distribution. $^{*}P$<0.05, $^{**}P$<0.01, $^{***}P$<0.001, analyzed by t-test. **b** Quantification of western blot analyses shown in Fig. 4f using ImageJ software. Data is presented as mean ± SEM, analyzed by t-test. $^{*}P$<0.05, $^{**}P$<0.01.

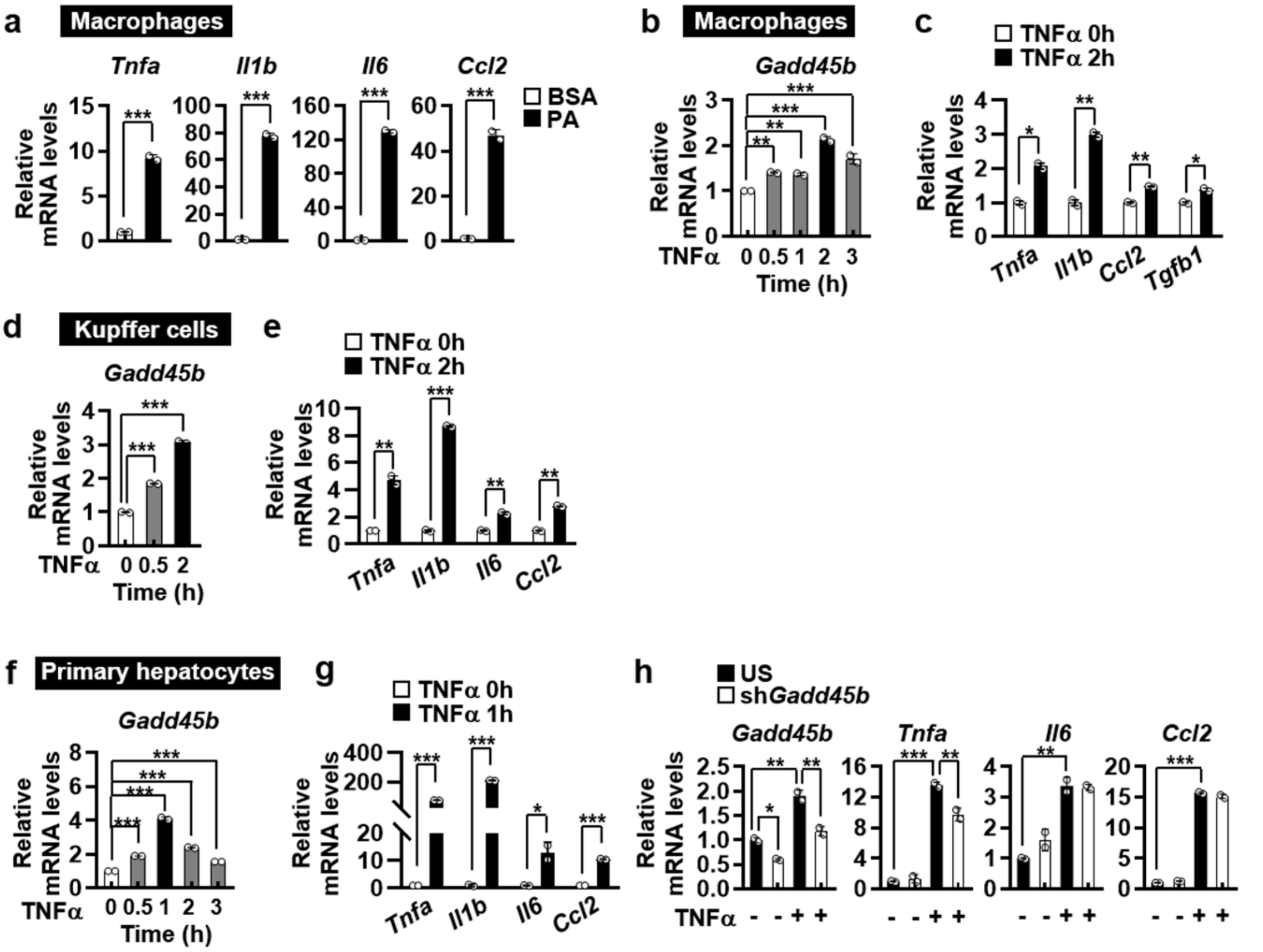

Supplementary Fig. 9. Changes in expression of *Gadd45b* and proinflammatory genes by fatty acids or TNFα treatment in macrophages, Kupffer cells, and hepatocytes.

a RT-qPCR analysis of inflammatory markers in RAW264.7 cells treated with BSA or 500 μM palmitic acid (PA) for 24 h. **b** RT-qPCR analysis of *Gadd45b* mRNA level in RAW264.7 cells treated with 50 ng/ml TNFα over time. **c** RT-qPCR analysis of inflammatory markers and *Tgfb1* in RAW264.7 cells treated with 50 ng/ml TNFα for 2 h. **d** RT-qPCR analysis of *Gadd45b* mRNA level in Kupffer cells treated with 50 ng/ml TNFα over time. **e** RT-qPCR analysis of inflammatory markers in Kupffer cells treated with 50 ng/ml TNFα for 2 h. **f** RT-qPCR analysis of *Gadd45b* mRNA level in primary hepatocytes treated with 10 ng/ml TNFα over time. **g** RT-qPCR analysis of inflammatory markers in primary hepatocytes treated with 10 ng/ml TNFα for 1 h. **h** RT-qPCR analysis showing the effects of *Gadd45b* knockdown on *Tnfa*, *Il6* and *Ccl2* mRNA levels in primary hepatocytes. Cells infected with Ad-US or Ad-sh*Gadd45b* were treated with or without 10 ng/ml TNFα for 1 h. Data in (**a, c, e** and **g**) are presented as mean ± SD, analyzed by t-test, and data in (**b, d, f** and **h**) are presented as mean ± SD, analyzed by one-way ANOVA followed by Tukey's test. $^{*}P<0.05$, $^{**}P<0.01$, $^{***}P<0.001$.

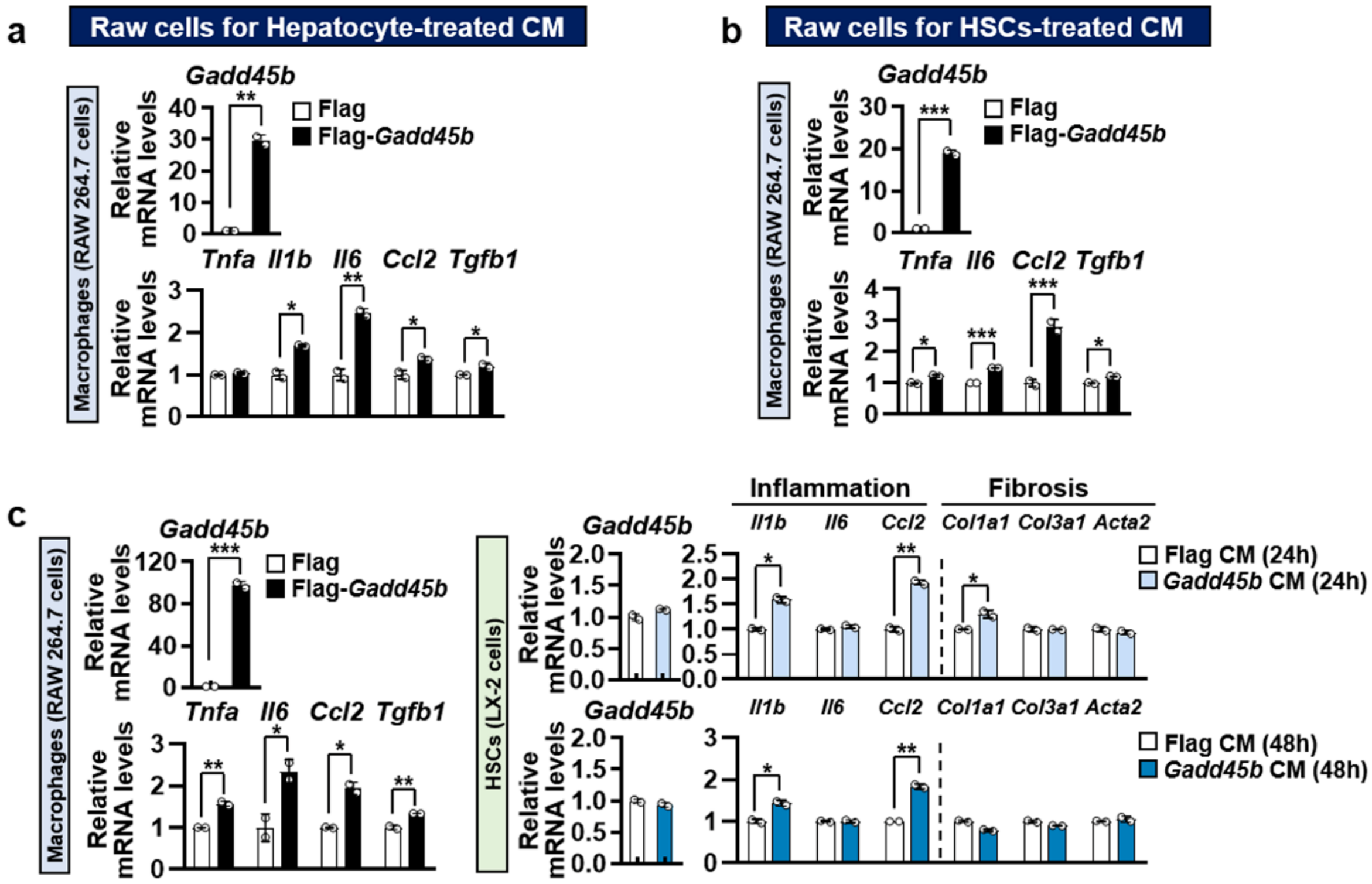

Supplementary Fig. 10. Effects of macrophage *Gadd45b* overexpression on Gadd45b and inflammatory gene expression in macrophages and LX-2 cells.

a, b RT-qPCR analysis showing the effects of *Gadd45b* overexpression on the mRNA levels of inflammatory genes and *Tgfb1* in RAW264.7 cells used to generate CM for subsequent treatment of hepatocytes (**a**) and HSCs (**b**). **c** RT-qPCR analysis showing the effects of *Gadd45b* overexpression on the mRNA levels of inflammatory genes and *Tgfb1* in RAW264.7 cells used to generate CM (left), and the effects of CM derived from RAW264.7 cells on the expression of inflammatory and fibrotic markers in LX-2 cells (right). Data in (**a–c**) are presented as mean ± SD, analyzed by t-test. $^{*}P$<0.05, $^{**}P$<0.01, $^{***}P$<0.001.

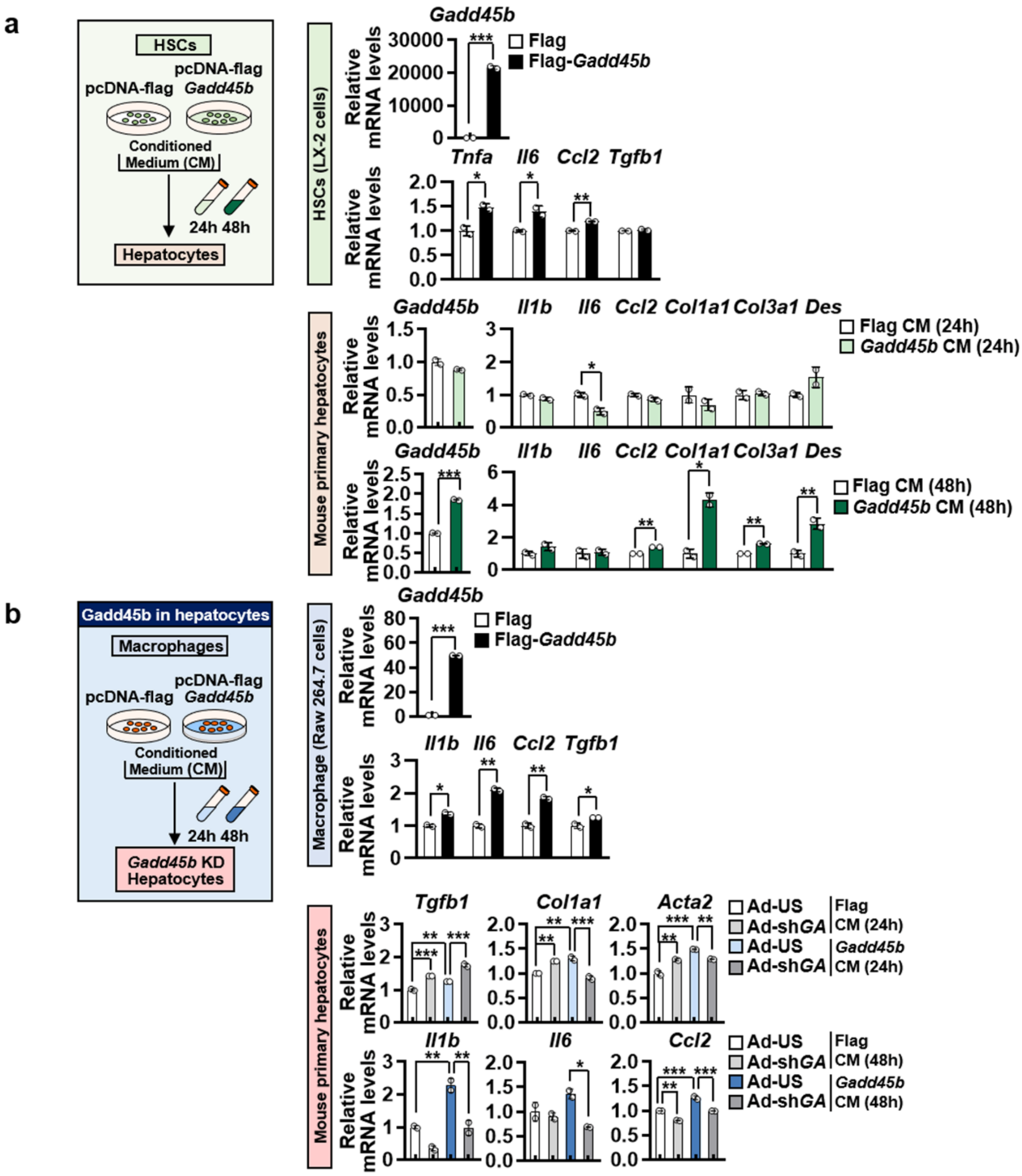

Supplementary Fig. 11. Effects of conditioned media from *Gadd45b*-overexpressing HSCs and macrophages on the expression of genes associated with inflammation and fibrosis in primary hepatocytes.

a Schematic diagram of the experimental design. CM were collected from the supernatants of LX-2 cells transfected with either pcDNA3-flag or pcDNA3-flag-*Gadd45b*. Mouse primary hepatocytes were subsequently cultured with the CM for 24 or 48 h. RT-qPCR analysis showing the effects of *Gadd45b* overexpression on the mRNA levels of inflammatory genes in LX-2 cells used to generate CM (top), and the effects of CM derived from LX-2 cells on the expression of inflammatory and fibrotic markers in mouse primary hepatocytes (bottom). **b** RT-qPCR analysis showing the effects of *Gadd45b* overexpression on the mRNA levels of inflammatory genes and *Tgfb1* in RAW264.7 cells used to generate CM (top), and the effects of *Gadd45b* knockdown on the mRNA levels of fibrotic or inflammatory markers in mouse primary hepatocytes treated with RAW264.7 cell-derived CM for 24 or 48 h (bottom). Primary

hepatocytes were infected with either Ad-US control or Ad-sh*Gadd45b* before CM treatment. Data in (**a**) and (**b**, top panel) are presented as mean ± SD, analyzed by t-test, and data in (**b**, bottom panel) is presented as mean ± SD, analyzed by one-way ANOVA followed by Tukey's test. $^{*}P<0.05$, $^{**}P<0.01$, $^{***}P<0.001$.

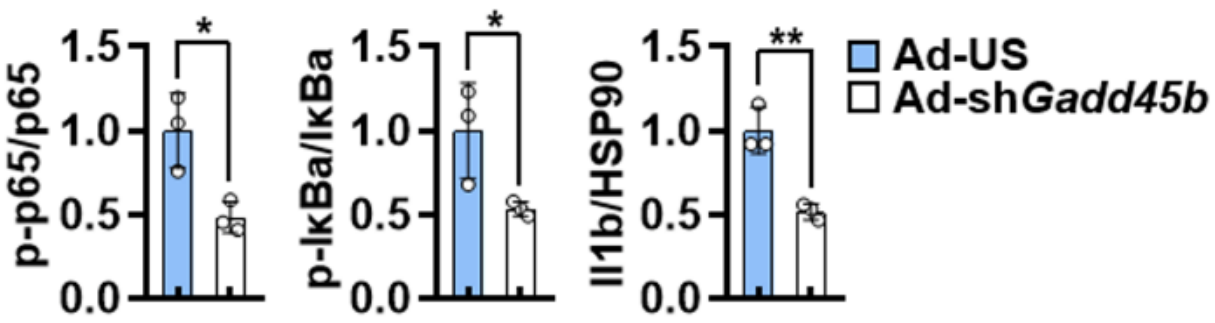

Supplementary Fig. 12. Effects of *Gadd45b* deficiency on TNFa-NFkB signaling under chronic HFD conditions

Quantification of western blot analyses shown in Fig. 5g using ImageJ software. Data is presented as mean ± SEM, analyzed by t-test. $^{*}P<0.05$, $^{**}P<0.01$.

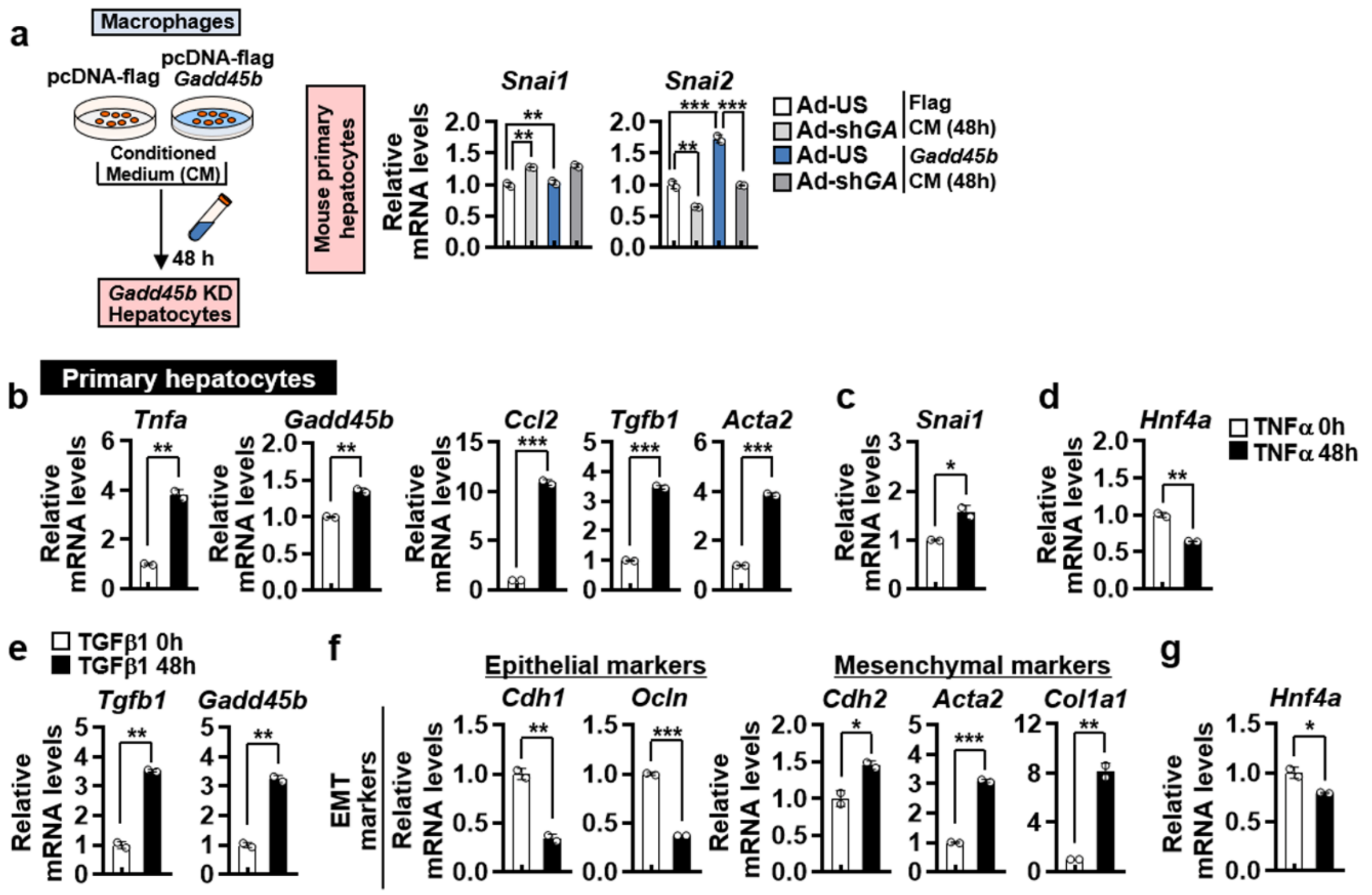

Supplementary Figure 13. Effects of CM from *Gadd45b*-expressing macrophages on inflammatory, fibrogenic, and EMT-related genes in primary hepatocytes.

a RT-qPCR analysis of the effects of *Gadd45b* knockdown on the mRNA levels of EMT-associated transcription factors in mouse primary hepatocytes treated with RAW264.7 cell-derived CM for 48 h. Primary hepatocytes were infected with either Ad-US control or Ad-sh*Gadd45b* before CM treatment. **b–d** RT-qPCR analysis of mRNA levels of *Tnfa*, *Gadd45b*, *Ccl2*, *Tgfb1*, *Acta2* (**b**), *Snai1* (**c**), and *Hnf4a* (**d**) in mouse primary hepatocytes treated with 10 ng/ml TNFα for 48 h. **e–g** RT-qPCR analysis of mRNA levels of *Tgfb1*, *Gadd45b* (**e**), EMT-related genes (**f**), and *Hnf4a* (**g**) in mouse primary hepatocytes treated with 5 ng/ml TGFβ1 for 48 h. Data in (**a**) is presented as mean ± SD, analyzed by one-way ANOVA followed by Tukey's test, data in (**b–g**) are presented as mean ± SD, analyzed by t-test. $^{*}P<0.05$, $^{**}P<0.01$, $^{***}P<0.001$.

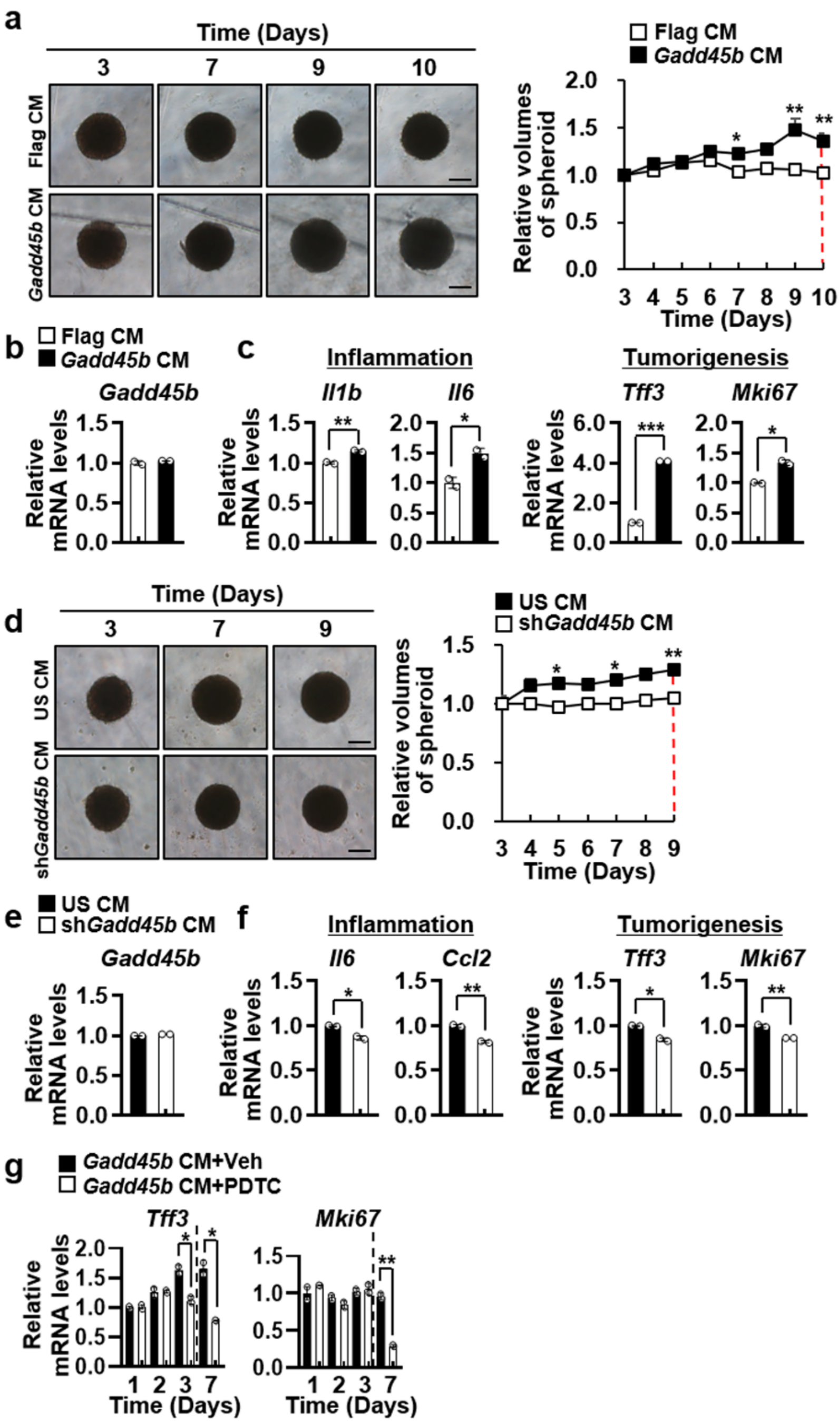

Supplementary Figure 14. Effects of CM from Gadd45b-expressing macrophages and PDTC on spheroid formation and progression.

a–c Huh7 spheroids were treated with CM derived from RAW264.7 cells expressing either Flag or Flag-*Gadd45b*. **a** Representative brightfield images of spheroids on days 3, 7, 9, and 10 (scale bar = 200 µm). The graph shows the change in spheroid volumes in Flag CM (5 spheroids) or *Gadd45b* CM (4 spheroids). **b, c** RT-qPCR analysis of mRNA levels of *Gadd45b* (**b**), inflammatory and tumorigenesis markers (**c**) in spheroids at day 10. **d–f** Huh7 spheroids were treated with CM derived from RAW264.7 cells expressing either US or U6-sh*Gadd45b*. **d** Representative brightfield images of spheroids on days 3, 7, and 9 (scale bar = 200 µm). The graph shows the change in spheroid volumes in US CM (5 spheroids) or sh*Gadd45b* CM (6 spheroids). **e, f** RT-qPCR analysis of mRNA levels of *Gadd45b* (**e**), inflammatory and tumorigenesis markers (**f**) in spheroids at day 9. **g** Huh7 spheroids treated with *Gadd45b* CM

derived from RAW264.7 cells were additionally treated with PDTC, which was replenished together with CM every 2 days. RT-qPCR analysis of mRNA levels of tumorigenesis markers in spheroids at the indicated time points. Data in (**a, c, d and f**) are presented as mean ± SD, analyzed by t-test, and data in (**g**) is presented as mean ± SD, analyzed by one-way ANOVA followed by Tukey's test. $^{*}P < 0.05$, $^{**}P < 0.01$, $^{***}P < 0.001$.

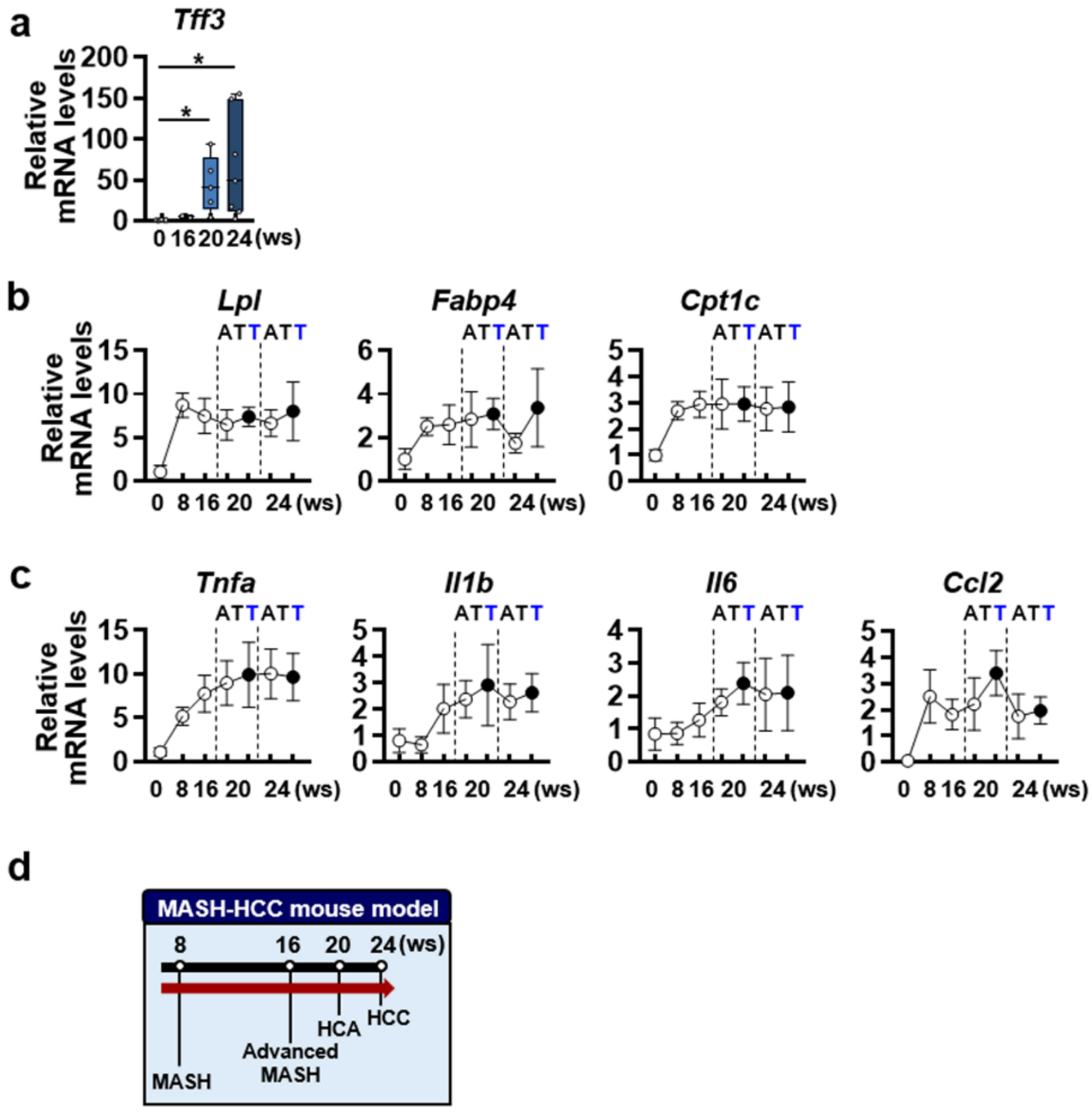

Supplementary Fig. 15. Expression pattern of the Lpl-Fabp4-Cpt1c axis and the TNFa-NFkB target genes during the progression of MASH to HCC in the livers of C57BL6N male mice.

a–**c** Tumor development mouse model: Groups include mice treated with MASH diet + CCl_4 for 0 (n = 6), 8 (n = 9), 16 (n = 5), 20 (n = 5) and 24 weeks (n = 7). **a** RT-qPCR analysis of *Tff3* mRNA levels in tumor tissues from the MASH diet + CCl_4 mice at 0 (n = 6), 16 (n = 2), 20 (n = 5) and 24 weeks (n = 7) (left) and schematic diagram showing the lesion stages of the MASH-HCC mouse model (right). The box plots show data distribution. $^{*}P<0.05$, analyzed by one-way ANOVA. **b, c** RT-qPCR analysis of MASH derived HCC markers (**b**) and TNFa-NFkb target genes (**c**) at 0, 8 and 16 weeks, as well as in adjacent nontumor and tumor tissue at 20 and 24 weeks. **d** Schematic diagram of the MASH, advanced MASH, HCA, and HCC phase in a MASH-HCC mouse model.

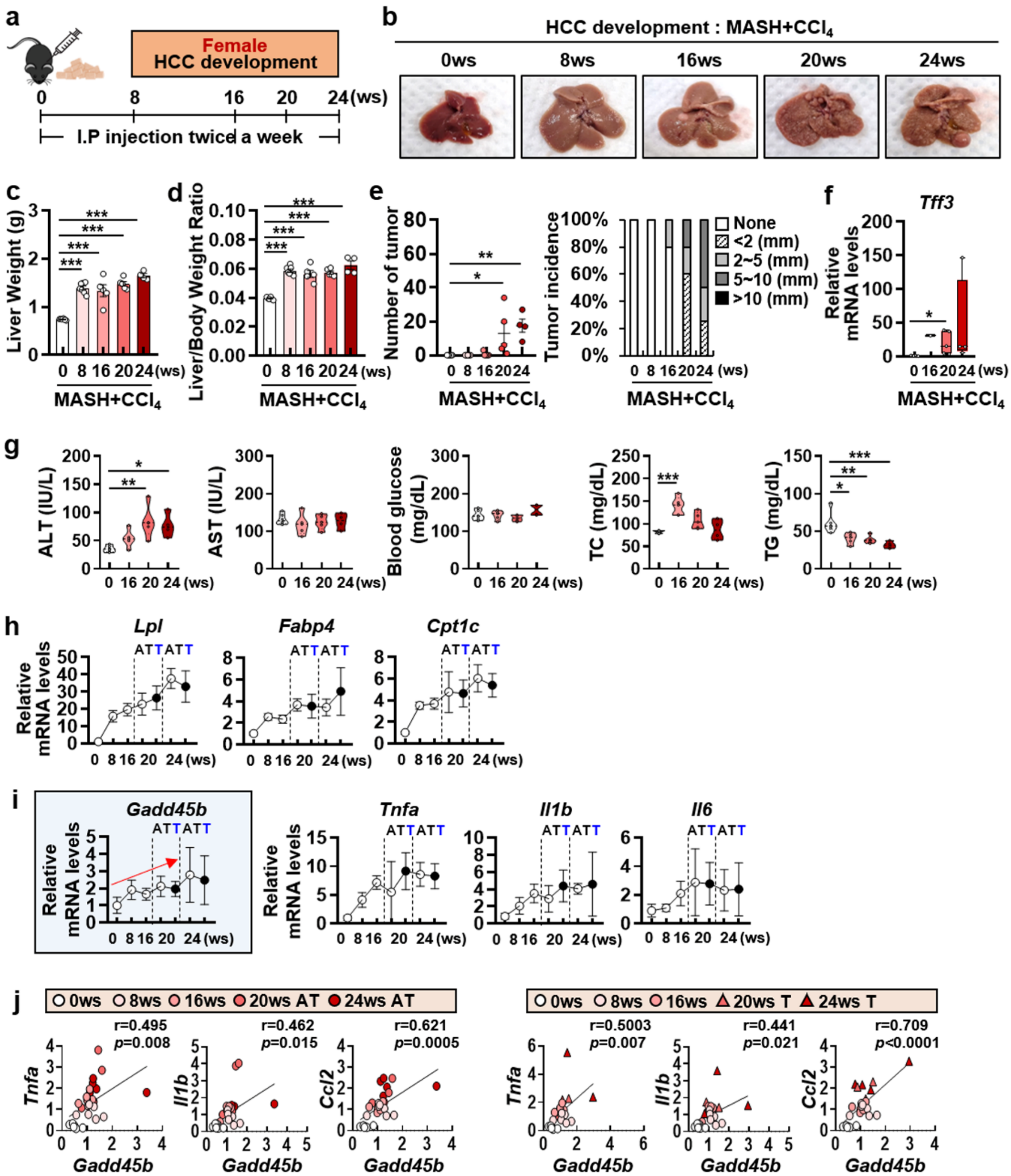

Supplementary Fig. 16. Gadd45b as a proinflammatory marker of MASH-to-HCC progression in C57BL6N female mice.

a Experimental design to determine lesion stages and validate proinflammatory Gadd45b as biomarker in the progression of MASH to HCC in female mouse model: Groups include mice treated with MASH diet + CCl_4 for 0 (n = 6), 8 (n = 7), 16 (n = 5), 20 (n = 5) and 24 weeks (n = 4). **b** Representative gross appearance of mouse livers. **c** Liver weight. **d** Liver per body weight ratio. **e** The number of visible tumors ≥ 1.0 mm per mouse and the percentages of mice with the indicated maximum tumor sizes. **f** RT-qPCR analysis of *Tff3* mRNA levels in tumor tissues from the MASH diet + CCl_4 mice at 0 (n = 6), 16 (n = 1), 20 (n = 5) and 24 weeks (n = 4). The box plots show data distribution. $^{*}P<0.05$, analyzed by one-way ANOVA. **g** Violin plots showing serum ALT, AST, blood glucose, total cholesterol (TC) and triglyceride (TG)

levels measured at 0, 16, 20 and 24 weeks. The thick dashed line and thin dotted line represent the median and interquartile range. $^{*}P<0.05$, $^{**}P<0.01$, $^{***}P<0.001$, analyzed by one-way ANOVA followed by Tukey's test. **h, i** RT-qPCR analysis of MASH derived HCC markers (**h**), *Gadd45b* and TNFa-NFkb target genes (**i**) at 0, 8 and 16 weeks, as well as in adjacent nontumor and tumor tissue at 20 and 24 weeks. **j** Pearson correlation graphs showing the correlation between *Gadd45b* expression and the expression levels of proinflammatory factors at 0, 8 and 16 weeks, as well as in adjacent nontumor and tumor tissue at 20 and 24 weeks. Pearson's correlation coefficients (r) and p-values are represented for each analysis. Data in **c**–**e** are presented as mean ± SEM, analyzed by one-way ANOVA followed by Tukey's test. $^{*}P<0.05$, $^{**}P<0.01$, $^{***}P<0.001$.

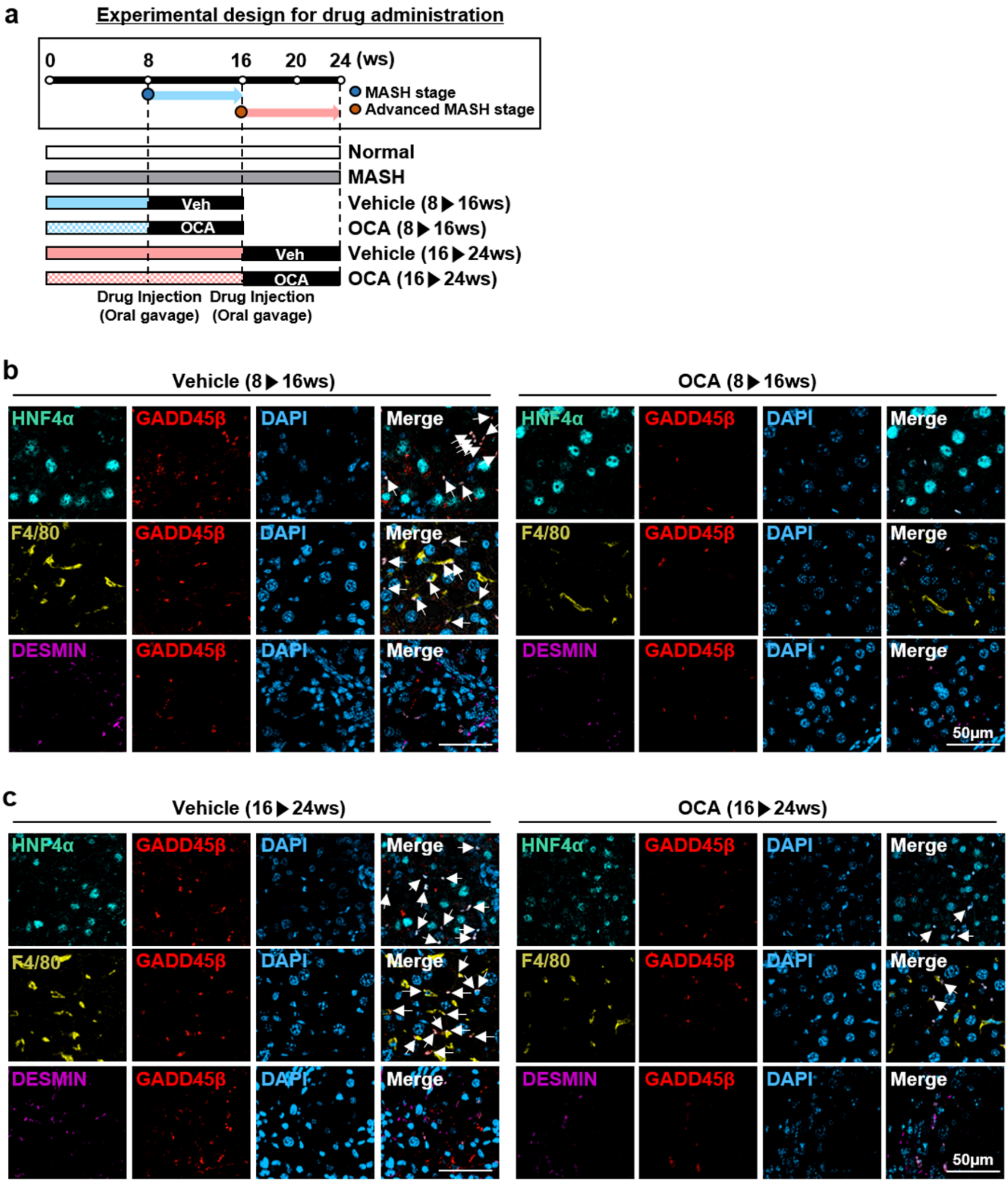

Supplementary Fig. 17. OCA reduces Gadd45b expression in hepatocytes and macrophages in MASH and advanced MASH livers.

a Experimental design for drug administration in MASH-HCC mouse model: Groups include the normal (*n* = 6), MASH (*n* = 8), vehicle-treated (*n* = 5), or OCA-treated mice (*n* = 7) from 8 to 16 weeks, and vehicle-treated (*n* = 4) or OCA-treated mice (*n* = 5) from 16 to 24 weeks. **b, c** Immunofluorescence staining of liver sections from mice administered vehicle or OCA for 8-16 weeks (**b**), or 16-24 weeks (**c**). Representative images showing co-localization of HNF4α (cyan), F4/80 (yellow), DESMIN (magenta), and Gadd45β (red). Nuclei were counterstained with DAPI (blue) (200×, scale bar = 50 μm).

Supplementary Table 1. Primer sequences for qPCR.

Gene	Forward (5’-3’)	Reverse (5’-3’)
m*L32*	GGCCTCTGGTGAAGCCCAAGATCG	CCTCTGGGTTTCCGCCAGTTTCGC
m*Gpc3*	CAACACTACCGACCACCTCAAG	ATCTCCACCACACCAGCCATAC
m*Lpl*	GGGTCACCTGGTCGAAGTAT	CCGAGTCCTCTCTCTGCAAT
m*Fabp4*	GTCACCATCCGGTCAGAGAG	TCGACTTTCCATCCCACTTC
m*Cpt1c*	GAAGGCAGGACAGAGACAGT	CTTCAGTAGAGCCTGGTGCT
m*Hnf4a*	AGAGGTTCTGTCCCAGCAGA	ATGTACTTGGCCCACTCGAC
m*Arg1*	TGACAACCAGCTCTGGGAAT	CGCAAGCCAATGTACACGAT
m*Mki67*	AGAGCACTCAACAGCAAAAGCC	TGCTAAAGAGCGCAAAGCCC
m*Tff3*	GAAGCTTGCCTGCTGCCATG	GCTTCAAAATGTGCATTCTG
m*Krt19*	TTCAGTACGCATTGGGTCAG	GGAGGACGAGGTCACGAA
m*Krt7*	TGAGATTGCGGAGATGAACC	CGATGCTGGACTCTAACTTGG
m*Epcam*	CCCTACGACCATCAGAGCTT	GTCCACGTCGTCTTGTGTTT
m*Ncam1*	CCAGGATTCCCAGTCCATGT	GCAACTGACCATCTCGGAAC
m*Cd36*	GCAAAGAAGGAAAGCCTGTG	CCAATGGTCCCAGTCTCATT
m*Fasn*	CCCTTGATGAAGAGGGATCA	GAACAAGGCGTTAGGGTTGA
m*Acaca*	CTCCAGGACAGCACAGATCA	TGACTGCCGAAACATCTCTG
m*Tnfa*	GTCCCCAAAGGGATGAGAAG	CACTTGGTGGTTTGCTACGA
m*Il1b*	ACCCTGCAGCTGGAGAGTGT	TTGACTTCTATCTTGTTGAAGACAAACC
m*Il6*	GGGAAATCGTGGAAATGAGAAAA	TGGTAGCATCCATCATTTCTTTG
m*Ccl2*	AGGTCCCTGTCATGCTTCTG	GCTGCTGGTGATCCTCTTGT
m*Adgre1*	CATCAGCCATGTGGGTACAG	AGTGTAGGAATCCCGCAATG
m*Tgfb1*	TGACGTCACTGGAGTTGTACGG	GGTTCATGTCATGGATGGTGC
m*Col1a1*	CCTGGCAAAGACGGACTCAAC	GCTGAAGTCATAACCGCCACTG
m*Gadd45a*	CGAAGACGACGACCGGGATG	TGTGGGTTCGTCACCAGCAC
m*Gadd45b*	CTTCACCCTGATCCAGTCGT	TGACAGTTCGTGACCAGGAG
m*Desmin*	CTAAAGGATGAGATGGCCCG	GAAGGTCTGGATAGGAAGGTTG
m*Acta2*	TGCTGTCCCTCTATGCCTCT	GAAGGAATAGCCACGCTCAG
m*Col3a1*	CTGTAACATGGAAACTGGGGAAA	CCATAGCTGAACTGAAAACCACC
m*Timp1*	ATTCAAGGCTGTGGGAAATG	CTCAGAGTACGCCAGGGAAC
m*Twist1*	CGGACAAGCTGAGCAAGATT	ATCCTCCAGACGGAGAAGG
m*Snail1*	CTTGTGTCTGCACGACCTGT	CTTCACATCCGAGTGGGTTT
m*Snail2*	CCTTTCTCTTGCCCTCACTG	ACAGCAGCCAGACTCCTCAT
m*Vimentin*	GACCTTGAACGGAAAGTGGA	AGCCACGCTTTCATACTGCT
m*Ocln*	TCAGGGAATATCCACCTATCACTTCAG	CATCAGCAGCAGCCATGTACTCTTCAC
m*Cdh1*	ACTGTGAAGGGACGGTCAAC	TGTCCCGGGTATCATCATCT
m*Cdh2*	TGTGGGAATCCGACGAATGGATGA	TGGAGCCACTGCCTTCATAGTCAA
h*Gadd45b*	GGGTGTACGAGTCGGCCAAG	CCGTGTGAGGGTTCGTGACC
h*Tnfa*	CCCAGGGACCTCTCTCTAATC	ATGGGCTACAGGCTTGTCACT
h*Il1b*	ACGCTCCGGGACTCACAGCA	TGAGGCCCAAGGCCACAGGT
h*Il6*	GGCACTGGCAGAAAACAACC	GCAAGTCTCCTCATTGAATCC
h*Ccl2*	CTCAGCCAGATGCAATCAATG	AGATCACAG CTTCTTTGGGACAC
h*Tff3*	CAGCTCTGCTGAGGAGTACG	AGGGATCCTGGAGTCAAAGC
h*Mki67*	TCAGGGAAGACCACACAGAC	TTGGGCCTCTTCCTTAGGTG
h*Cdh1*	TGAAGGTGACAGAGCCTCTGGA	TGGGTGAATTCGGGCTTGTT
h*Cdh2*	GCGTGTGAAGGTTTGCCAGTG	CCGGCGTTTCATCCATACCACAA
h*Vimentin*	TGGCCGACGCCATCAACACC	CACCTCGACGCGGGCTTTGT
h*Acta2*	TTCAATGTCCCAGCCATGTA	GAAGGAATAGCCACGCTCAG

Supplementary Table 2. Antibodies involved in this study.

Antibody	Company	Catalog number
**Gpc3**	Thermo Fisher, USA	MA5-17083
**Hnf4a**	Abcam, USA	ab201460
**Ki67**	Thermo Fisher, USA	14-5698-82
**Ck19**	Abcam, USA	ab52625
**F4/80**	Cell Signaling Technology, USA	70076S
**GADD45β**	Santa Cruz Biotechnology, USA	sc-8776
**Desmin**	Cell Signaling Technology, USA	73348S
**Phospho-NF-κB p65**	Cell Signaling Technology, USA	#3033
**NF-κB p65**	Cell Signaling Technology, USA	#4764
**Phospho-IκBα**	Cell Signaling Technology, USA	#9246
**IκBα**	Cell Signaling Technology, USA	#4812
**IL-1β**	Cell Signaling Technology, USA	#31202
**HSP90**	Santa Cruz Biotechnology, USA	sc-13119

Supplementary Table 3. Characterization of liver tissue histopathology.

Group / Characterization	NCD	MASH+CCl_4^{high}	
	**Normal**	**Adjacent nontumor**	**Tumor**
**Relationship to adjacent liver parenchyma**	–	Well demarcated	Well demarcated
**Pleomorphism**	–	+	+
**Steatosis**	–	++	++
**Hepatocyte ballooning**	–	√	√
**Mallory-denk bodies**	–	√	√
**Inflammation**	–	++	+
**Fibrosis**	–	++	–
**Reticulin fiber**	–	++	+
**Portal tract**	√	√	–
**Ductular reaction**	–	√	–

(–: absence, √: presence, +: mild, ++: marked)
